# Modelling the dark side of prison dynamics: Quantifying the criminogenic effects of petty crime offenders’ incarceration

**DOI:** 10.1371/journal.pone.0356366

**Published:** 2026-09-16

**Authors:** Farai Nyabadza

**Affiliations:** Department of Mathematics and Applied Mathematics, University of Johannesburg, Johannesburg, South Africa; University of Padova: Universita degli Studi di Padova, ITALY

## Abstract

Crime remains a persistent socio-economic challenge in South Africa, where the incarceration of petty offenders often exacerbates criminal behaviour. Empirical evidence suggests custodial sentencing for minor offences may accelerate progression to serious crime due to exposure to hardened offenders and criminogenic prison conditions. To investigate this, we develop a compartmental ODE model tracking eight population states: susceptibility, petty and serious crime, custodial and non-custodial sentencing, rehabilitation, and relapse pathways. In particular, the model has an explicit non-custodial sentencing compartment, positioned in a distinct community-supervision stratum that is neither the general community nor the prison system, enabling direct comparison of custodial and non-custodial policy scenarios. The model is parameterised using the South African crime and prison data. Sensitivity analysis shows that sentencing rates, rehabilitation efficacy, and relapse probabilities drive long-term dynamics. Numerical simulations reveal that over-reliance on custodial sentencing perpetuates recidivism and incarceration, while prioritising non-custodial disposals and rehabilitation reduces both ${ℛ}_{0}$ and CCR. Findings indicate that indiscriminate incarceration of petty offenders entrenches criminal cycles. Evidence-based interventions focused on rehabilitation and relapse prevention offer durable crime reduction. This quantitative framework evaluates justice policies, highlighting reforms that integrate deterrence with effective rehabilitation.

## Introduction

Recent data from the South African Police Service (SAPS) for the 2022/2023 reporting period document a marked escalation in violent criminal activity, characterised by a substantial rise in cash-in-transit heists [1]. Concurrently, a surge in mass shooting incidents underscores the intensification of high-impact violent crime across the country [2]. While these phenomena attract the greatest public and policy attention, petty crimes remain pervasive and exert a substantial influence on the broader criminological landscape [3]. SAPS statistics for the same period indicate that petty offences, primarily theft and burglary, accounted for between 60% and 75% of all reported criminal cases, while serious crimes, including aggravated robbery and homicide, continued to impose a heavy societal burden, with more than 27,000 homicides recorded in 2022/2023 [1].

Crime, encompassing both minor (petty) and major (serious) offences, constitute a multifaceted and enduring challenge to global societal stability. Petty crimes, typically classified as low-severity infractions, include acts such as shoplifting, vandalism, and possession of small quantities of illicit substances [4]. Serious crimes, by contrast, involve high-severity offences such as armed robbery, assault, and homicide [5]. Within the South African context, both categories are highly prevalent and together contribute to elevated public safety concerns and deepening doubts regarding the efficacy of the criminal justice system.

Incarceration has historically been justified on the grounds of rehabilitation and deterrence [6], yet accumulating evidence challenges its effectiveness. Research suggests that imprisonment may not only fail to reduce crime but can, under certain conditions, intensify criminal behaviour [7]. In South Africa, chronically overcrowded prisons routinely house minor offenders alongside hardened criminals [8,9], creating criminogenic environments that substantially elevate recidivism risk [10]. This dynamic is consistent with the Prison Industrial Complex thesis, which holds that the carceral system sustains, rather than disrupts, cycles of criminality within broader socio-economic structures [11].

Mathematical modelling, particularly through compartmental systems of ordinary differential equations, provides a powerful and tractable framework for analysing the dynamics of criminal behaviour [12,13]. Such models partition the population into distinct subgroups and specify transition rates between compartments, thereby enabling simulation of crime progression and systematic evaluation of policy interventions [12]. Building on the foundational economic theories of crime and deterrence due to Becker [4] and Ehrlich [14], compartmental crime models incorporate deterrence mechanisms, rehabilitation processes, and socio-economic influences. More recent extensions have incorporated incarceration-induced behavioural change and recidivism dynamics [15].

Significant progress in mathematical modelling has shed light on the mechanisms through which incarceration shapes criminal behaviour. Chakra and Hilbe [16] developed a compartmental model demonstrating that prolonged incarceration can escalate serious criminality through prison socialisation. Accinelli et al. [17] applied predator-prey dynamics, framing incarceration as “predation” and establishing that excessive imprisonment yields diminishing deterrence returns. Kwofie et al. [18] integrated rehabilitation explicitly, showing that balanced investment in both incarceration and rehabilitation achieves optimal crime control. Collectively, these studies highlight the nonlinear and often counterproductive consequences of exclusively punitive policies [12].

The present study advances the existing literature in three key respects. First, unlike Abou Chakra and Hilbe [16], who model escalation without deriving a CCR threshold or incorporating a non-custodial pathway, we introduce the compartment $A_{p}$ as a distinct supervised probation excluded from crime initiation, together with the associated CSER metric $\Psi_{pn}$, to enable direct comparison of sentencing regimes. Second, in contrast to Accinelli et al. [17], whose predator-prey framework yields neither a crime generation number nor a carceral criminality threshold, we derive both ${ℛ}_{0}$, $\Phi_{\mathrm{pn}}^{\left(a\right)}$, and $\Psi_{pn}$ analytically. Third, unlike Kwofie et al. [18], who include rehabilitation but do not distinguish custodial from non-custodial pathways, our model facilitates evaluation of differential criminogenic risk by sentencing type. Recent complementary contributions include compartmental models with optimal control [19], recidivism-focused frameworks [20] and fractional-order extensions [21].

The contagion analogy employed in this model rests on three complementary foundations. First, an extensive body of empirical criminological research documents strong peer-influence and social-learning dynamics in the onset and persistence of criminal behaviour, particularly among urban youth. These dynamics are structurally analogous to the transmission of infectious disease [15,22–25]. The criminal career literature [15] further demonstrates that offending is both initiated and sustained through social exposure, directly mirroring the epidemiological force-of-infection concept. Second, the chronically overcrowded South African prison environment, which places petty offenders in sustained contact with serious offenders [8,9], generates a “super-spreader” dynamic that is captured explicitly by the carceral escalation pathway in the model. Third, the contagion analogy necessarily abstracts away individual agency, economic incentives [4,14], and structural drivers such as poverty and unemployment, each of which is acknowledged explicitly in the Limitations section.

Building on this body of evidence, the present study addresses a gap in the literature by constructing a model that simultaneously tracks custodial and non-custodial sentencing pathways, derives explicit escalation thresholds, and calibrates findings to South African data. This study is motivated by the need to evaluate the systemic consequences of incarcerating petty offenders in South Africa. The primary objective is to quantify the effects of sentencing and rehabilitation policies on long-run crime dynamics through the derivation and analysis of three threshold parameters: the crime generation number (${ℛ}_{0}$), the CCR ($\Phi_{pn}^{\left(a\right)}$), and the community-supervision escalation ratio (CSER, $\Psi_{pn}$). Through global sensitivity analysis and numerical simulation, we assess the impact of a range of policy interventions, with particular attention to the roles of non-custodial sentencing and structured rehabilitation in mitigating recidivism and reducing aggregate criminal activity.

Throughout this paper, we use the pairing petty and serious as the primary descriptive terms for the two offence-severity categories, so that the severity ordering is transparent to readers across jurisdictions; “petty” and “serious” are descriptive rather than statutory categories in South African law, which distinguishes offences by schedule [26] and by Magistrates’/High Court jurisdiction [27].

## Materials and methods

### Mathematical model

The compartmental model describes the population dynamics of individuals transitioning among states defined by criminal behaviour and rehabilitation status. The model comprises eight compartments: susceptibles (*S*); active petty offenders ($C_{p}$); active serious (serious) offenders ($C_{n}$); petty offenders serving non-custodial sentences ($A_{p}$); petty offenders serving custodial sentences ($J_{p}$); serious offenders serving custodial sentences ($J_{n}$); individuals rehabilitated from a petty-crime sentence ($R_{p}$); and individuals rehabilitated from a serious-crime custodial sentence ($R_{n}$). The compartments *S*, $C_{p}$, $C_{n}$, $R_{p}$, and $R_{n}$ describe community-level dynamics, while $J_{p}$ and $J_{n}$ represent the incarcerated (prison) population. The compartment $A_{p}$ occupies a distinct community-supervision stratum where individuals in $A_{p}$ are neither freely active in the general community nor detained in prison. They reside in the community under formal judicial supervision (e.g., community service, probation, electronic monitoring). It is important to clarify the meaning of the rehabilitated classes. $R_{p}$ denotes individuals who have completed either a custodial sentence via $J_{p}$ or a non-custodial sentence via $A_{p}$ for a petty offence and have re-entered the community without active criminal engagement. $R_{n}$ denotes individuals rehabilitated following a serious-crime custodial sentence (including petty offenders who escalated due to imprisonment in $J_{p}$). These classes are treated as distinct states because post-release reintegration outcomes, recidivism risk, and supervision requirements differ systematically between minor and serious offenders [10].

Rehabilitation classes are defined by offending severity at release rather than by the original offence. Individuals leaving at the petty-offending level enter $R_{p}$, whereas those leaving at the serious-offending level enter $R_{n}$. Thus, a petty offender may enter $R_{n}$ if escalation occurs during custody. This process is governed by the carceral escalation probability, π∈(0,1]: of offenders released from $J_{p}$ at rate $\rho_{p}$, a fraction π exits into $R_{n}$ due to prison-induced socialisation into serious offending, while the remaining fraction (1−π) exits into $R_{p}$. No such split occurs along the non-custodial pathway $A_{p}\to R_{p}$, since it bypasses the prison environment. Hence, π represents the probability that imprisonment itself generates a serious offender, irrespective of the initial offence. At the population level, this effect is captured by the carceral-induced criminality ratio (CCR, $\Phi_{pn}^{\left(a\right)}$), which incorporates π together with the probabilities of custodial sentencing and subsequent relapse into serious crime. The empirical justification for this escalation mechanism is provided by evidence of prison socialisation and overcrowded co-detention in South Africa, as discussed earlier in the Introduction and modelling assumptions. The total population of individuals in the community, those under custodial sentences and prison, is thus given by


$$\begin{array}{c} N(t)=S+C_{p}+C_{n}+A_{p}+J_{p}+J_{n}+R_{p}+R_{n}. \end{array}$$

The model is constructed under the following explicit assumptions. Criminal behaviour spreads through social contagion, analogous to the transmission of an infectious disease [22,23]. All individuals within a given compartment are assumed homogeneous; demographic and socio-economic heterogeneity within compartments is neglected. Rehabilitation is imperfect: individuals in $R_{p}$ and $R_{n}$ may relapse into criminal activity. There is no pre-trial detention delay; convicted individuals transition directly from $C_{p}$ or $C_{n}$ to the appropriate sentencing compartment, a standard assumption in first-generation compartmental crime models [12]. Individuals in the community-supervision compartment $A_{p}$ are under active judicial oversight and are therefore do not contribute to crime generation. This assumption reflects the empirical observation that supervised diversion programmes substantially curtail the unsupervised peer-influence contacts that drive criminal contagion [28,29]. Finally, the custodial pathways for petty and serious offenders remain distinct throughout sentencing; no transfer between $J_{p}$ and $J_{n}$ is permitted.

The escalation mechanism is grounded in documented conditions within the South African correctional system rather than being purely a modelling assumption. Although the Correctional Services Act 111 of 1998 provides for the classification and separation of offenders by risk level, persistent overcrowding, remand–sentenced mixing, and shared communal accommodation often undermine these provisions, resulting in sustained contact between petty and serious offenders [9,28–31]. This co-detention environment provides the empirical basis for the custody-induced escalation mechanism represented by π>0. In contrast, the prison compartments $J_{p}$ and $J_{n}$ remain separate accounting classes without administrative transfer, a simplification adopted for modelling tractability. In settings where petty and serious offenders are effectively segregated, the escalation parameter π would be expected to decrease, with the limiting case π→0 corresponding to a system in which imprisonment generates no criminogenic escalation.

The susceptible population is recruited at a constant rate Λ with a proportion q∈(0,1) of individuals committing petty offences, while the complementary proportion (1−q) commits serious offences upon recruitment into criminality driven by the force of crime transmission λ. Crime generation is assumed to be driven by actively offending individuals in the community and individuals in the community-supervision stratum $A_{p}$, the prison compartments $J_{p}$, $J_{n}$, and the rehabilitated classes $R_{p}$, $R_{n}$ do not directly generate new criminal contacts. So,


$$\begin{array}{c} \lambda =\beta (C_{p}+\eta \;C_{n}). \end{array}$$

Here, β denotes the effective contact rate and η≥1, captures the greater criminogenic influence of serious offenders relative to petty offenders. Among convicted petty offenders, a proportion κ∈(0,1) receive a non-custodial sentence, joining $A_{p}$ at rate $\sigma_{p}$, while the remainder (1−κ) receive a custodial sentence, joining $J_{p}$. All serious offenders receive custodial sentences and enter $J_{n}$ at rate $\sigma_{n}$. Individuals in $A_{p}$ complete their supervised sentence and transition to $R_{p}$ at rate $\rho_{a}$. Most importantly, this passage from $A_{p}$ to $R_{p}$ bypasses the prison environment entirely, so no carceral escalation can occur along this route. Individuals sentenced custodially and in compartment $J_{n},$ transition to $R_{n}$ upon release at rate $\rho_{n}$.

The transition from petty incarceration $J_{p}$ is modelled as a probabilistic escalation process. Upon release at rate $\rho_{p}$, a fraction π∈(0,1] of individuals is placed into the serious-offender rehabilitated class $R_{n}$, reflecting prison-induced escalation in offending severity, while the complementary fraction (1−π) transitions to the petty rehabilitated class $R_{p}$. The limiting case π=1 corresponds to a fully escalatory custodial environment, which is consistent with empirical evidence from the South African context [32,33].

Relapse from the rehabilitated classes is governed by four direct transition rates, each corresponding to a distinct pathway in the model diagram. To motivate the parameterisation, let $\omega_{p}$ and $\omega_{n}$ denote the aggregate relapse rates from $R_{p}$ and $R_{n}$, respectively, and let $\alpha_{p},\alpha_{n}\in [0,1]$ denote the cross-escalation probabilities. Within each rehabilitated class, a fraction of relapses returns the individual to the same offending level (own-class relapse), while the complementary fraction transitions to the other offending level (cross-class escalation or de-escalation). Specifically, the four pathway-specific rates are defined by


$$\begin{array}{c} \omega_{pp}=(1-\alpha_{p})\;\omega_{p},\;\omega_{pn}=\alpha_{p}\;\omega_{p},\;\omega_{nn}=(1-\alpha_{n})\;\omega_{n},\;\omega_{np}=\alpha_{n}\;\omega_{n}, \end{array}$$ (1)

so that the four rates admit the following interpretations; $\omega_{pp}=(1-\alpha_{p})\omega_{p}$ denotes the rate at which individuals in $R_{p}$ relapse to petty crime, $\omega_{pn}=\alpha_{p}\omega_{p}$ denotes the rate at which individuals in $R_{p}$ escalate to serious crime, $\omega_{nn}=(1-\alpha_{n})\omega_{n}$ denotes the rate at which individuals in $R_{n}$ relapse to serious crime, and $\omega_{np}=\alpha_{n}\omega_{n}$ denotes the rate at which individuals in $R_{n}$ de-escalate to petty crime. By construction, the decomposition (1) satisfies the aggregate consistency conditions


$$\begin{array}{c} \omega_{pp}+\omega_{pn}=\omega_{p},\;\omega_{nn}+\omega_{np}=\omega_{n}, \end{array}$$ (2)

ensuring that the total outflow rate from each rehabilitated class is preserved. The baseline configuration $\alpha_{p}=\alpha_{n}=0$ (equivalently, $\omega_{pn}=\omega_{np}=0$) corresponds to strictly class-specific relapse with no cross-pathway transitions. The escalation-dominant regime, $\alpha_{p}>0$ and $\alpha_{n}\approx 0$ (equivalently, $\omega_{pn}>0$ and $\omega_{np}\approx 0$), captures the empirically documented asymmetry in recidivism whereby upward transitions to more serious crimes substantially predominate over de-escalation [10,32].

**Fig 1:**
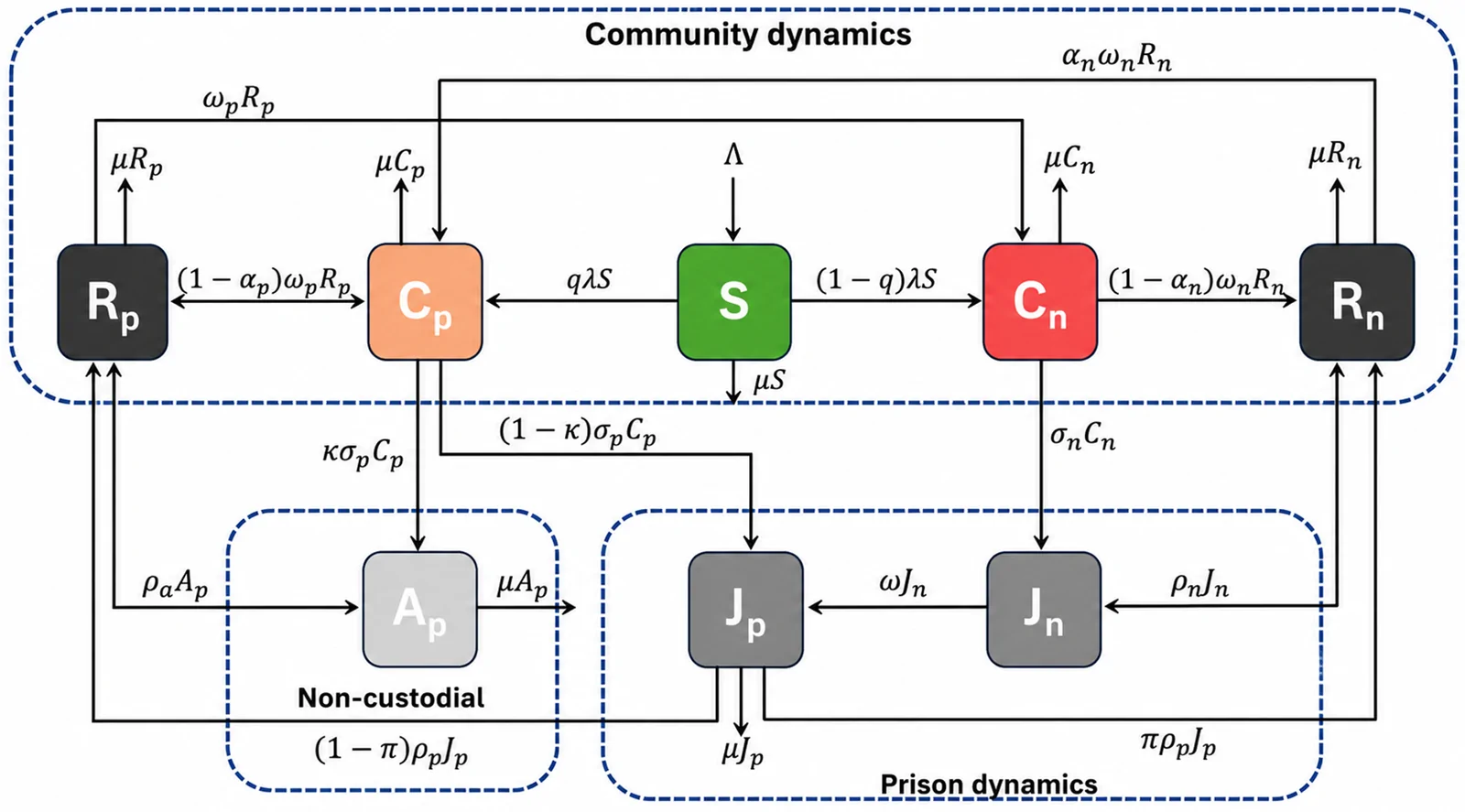
Flow diagram of the crime and rehabilitation model. The diagram illustrates the transitions between population compartments: Susceptibles (*S*), Criminals committing petty ($C_{p}$) or serious crimes ($C_{n}$), jailed individuals ($J_{p}$, $J_{n}$), those serving non-custodial sentences ($A_{p}$), and rehabilitated individuals ($R_{p}$, $R_{n}$). Arrows represent the movement between states, driven by the model’s parameters.

A flow diagram of the model is shown in Fig 1.

The dynamics of the model are governed by the following system of ordinary differential equations:


$$\begin{array}{ccc} \frac{dS}{dt} & = & \Lambda -\lambda S-\mu S, \end{array}$$ (3)

$$\begin{array}{ccc} \frac{dC_{p}}{dt} & = & q\lambda S+\omega_{pp}R_{p}+\omega_{np}R_{n}-Q_{1}\;C_{p}, \end{array}$$ (4)

$$\begin{array}{ccc} \frac{dC_{n}}{dt} & = & (1-q)\lambda S+\omega_{pn}R_{p}+\omega_{nn}R_{n}-Q_{2}\;C_{n}, \end{array}$$ (5)

$$\begin{array}{ccc} \frac{dA_{p}}{dt} & = & \kappa \sigma_{p}C_{p}-Q_{3}\;A_{p}, \end{array}$$ (6)

$$\begin{array}{ccc} \frac{dJ_{p}}{dt} & = & (1-\kappa )\sigma_{p}C_{p}-Q_{4}\;J_{p}, \end{array}$$ (7)

$$\begin{array}{ccc} \frac{dJ_{n}}{dt} & = & \sigma_{n}C_{n}-Q_{5}\;J_{n}, \end{array}$$ (8)

$$\begin{array}{ccc} \frac{dR_{p}}{dt} & = & \rho_{a}A_{p}+(1-\pi )\rho_{p}J_{p}-Q_{6}\;R_{p}, \end{array}$$ (9)

$$\begin{array}{ccc} \frac{dR_{n}}{dt} & = & \rho_{n}J_{n}+\pi \rho_{p}J_{p}-Q_{7}\;R_{n}, \end{array}$$ (10)

where the composite outflow rates are defined as


$$\begin{array}{c} Q_{1}=\mu +\sigma_{p},\;Q_{2}=\mu +\sigma_{n},\;Q_{3}=\mu +\rho_{a},\;Q_{4}=\mu +\rho_{p},\;Q_{5}=\mu +\rho_{n}, \end{array}$$

$$\begin{array}{c} Q_{6}=\mu +\omega_{pp}+\omega_{pn},\;Q_{7}=\mu +\omega_{nn}+\omega_{np}.\; \end{array}$$

The system is subject to the initial conditions


$$\begin{array}{c} S(0)=S_{0}>0,\;C_{p}(0)=C_{p0}\geq 0,\;C_{n}(0)=C_{n0}\geq 0,\;A_{p}(0)=A_{p0}\geq 0,\\ J_{p}(0)=J_{p0}\geq 0,\;J_{n}(0)=J_{n0}\geq 0,\;R_{p}(0)=R_{p0}\geq 0,\;R_{n}(0)=R_{n0}\geq 0. \end{array}$$ (11)

All parameters are assumed strictly positive unless otherwise stated.

## Model properties

### Positivity of solutions

**Theorem 1.**

*Let* $\left(S(t),C_{p}(t),C_{n}(t),A_{p}(t),J_{p}(t),J_{n}(t),R_{p}(t),R_{n}(t)\right)$ *be the solution of system (3)–(10) with positive initial conditions as given in (11). Then all components remain strictly positive for all t > 0.*

*Proof.*

We examine the vector field on the boundary of the non-negative orthant. For each compartment, when that compartment is zero and all other compartments are non-negative, the corresponding derivative is non-negative:


$$\begin{array}{cc} {\frac{dS}{dt}|}_{S=0} & =\Lambda \;>\;0,\;{\frac{dC_{p}}{dt}|}_{C_{p}=0}=q\lambda S+\omega_{pp}R_{p}+\omega_{np}R_{n}\;\geq \;0,\\ {\frac{dC_{n}}{dt}|}_{C_{n}=0} & =(1-q)\lambda S+\omega_{pn}R_{p}+\omega_{nn}R_{n}\;\geq \;0,\\ {\frac{dA_{p}}{dt}|}_{A_{p}=0} & =\kappa \sigma_{p}C_{p}\;\geq \;0,\;{\frac{dJ_{p}}{dt}|}_{J_{p}=0}=(1-\kappa )\sigma_{p}C_{p}\;\geq \;0,\;{\frac{dJ_{n}}{dt}|}_{J_{n}=0}=\sigma_{n}C_{n}\;\geq \;0,\\ {\frac{dR_{p}}{dt}|}_{R_{p}=0} & =\rho_{a}A_{p}+(1-\pi )\rho_{p}J_{p}\;\geq \;0,\;{\frac{dR_{n}}{dt}|}_{R_{n}=0}=\rho_{n}J_{n}+\pi \rho_{p}J_{p}\;\geq \;0. \end{array}$$

All right-hand sides are continuous and satisfy the quasi-positivity condition (i.e., whenever a variable is zero, its derivative is non-negative provided the other variables are non-negative). Since the vector field satisfies the quasi-positivity condition (each derivative is non-negative whenever the corresponding variable is zero and all other variables are non-negative), the non-negative orthant $R_{+}^{8}$ is positively invariant. Starting from strictly positive initial conditions, a standard continuity argument further implies that every component remains strictly positive for all *t* > 0. □

### Boundedness and Invariant Region

Let the feasible region for model (3)–(10) be


$$\begin{array}{c} \Omega =\left\{(S,C_{p},C_{n},A_{p},J_{p},J_{n},R_{p},R_{n})\in {\mathbb{R}}_{+}^{8}:N(t)\leq \frac{\Lambda }{\mu }\right\}. \end{array}$$

**Theorem 2.** *The feasible region* Ω *is positively invariant and attracting under the flow of system (3)–(10).*

*Proof.*

Summing Eqs (3)–(10), the dynamics of the total population satisfy


$$\begin{array}{c} \frac{dN}{dt}=\Lambda -\mu N. \end{array}$$

This is a linear first-order ODE whose solution is


$$\begin{array}{c} N(t)=\frac{\Lambda }{\mu }+\left(N(0)-\frac{\Lambda }{\mu }\right)e^{-\mu t}. \end{array}$$

It follows that N(t)≤Λ/μ whenever N(0)≤Λ/μ, establishing positive invariance of Ω. Moreover, N(t)→Λ/μ monotonically as t→∞, regardless of the initial value *N*(0), so Ω is attracting. This completes the proof.

Theorems 1 and 2 together establish that system (3)–(10) is mathematically well-posed: solutions exist, are unique, remain strictly positive, and are confined to the compact, epidemiologically meaningful domain Ω.

## Steady states

To determine the steady states, we set all crime-related state variables to zero and solve the resulting algebraic system. Setting the right-hand sides of Eqs (3)–(10) equal to zero gives


$$\begin{array}{ccc} 0 & = & \Lambda -\beta (C_{p}^{*}+\eta C_{n}^{*})S^{*}-\mu S^{*}, \end{array}$$ (12)

$$\begin{array}{ccc} 0 & = & q\beta (C_{p}^{*}+\eta C_{n}^{*})S^{*}+\omega_{pp}R_{p}^{*}+\omega_{np}R_{n}^{*}-Q_{1}C_{p}^{*}, \end{array}$$ (13)

$$\begin{array}{ccc} 0 & = & (1-q)\beta (C_{p}^{*}+\eta C_{n}^{*})S^{*}+\omega_{pn}R_{p}^{*}+\omega_{nn}R_{n}^{*}-Q_{2}C_{n}^{*}, \end{array}$$ (14)

$$\begin{array}{ccc} 0 & = & \kappa \sigma_{p}C_{p}^{*}-Q_{3}A_{p}^{*}, \end{array}$$ (15)

$$\begin{array}{ccc} 0 & = & (1-\kappa )\sigma_{p}C_{p}^{*}-Q_{4}J_{p}^{*}, \end{array}$$ (16)

$$\begin{array}{ccc} 0 & = & \sigma_{n}C_{n}^{*}-Q_{5}J_{n}^{*}, \end{array}$$ (17)

$$\begin{array}{ccc} 0 & = & \rho_{a}A_{p}^{*}+(1-\pi )\rho_{p}J_{p}^{*}-Q_{6}R_{p}^{*}, \end{array}$$ (18)

$$\begin{array}{ccc} 0 & = & \rho_{n}J_{n}^{*}+\pi \rho_{p}J_{p}^{*}-Q_{7}R_{n}^{*}, \end{array}$$ (19)

where


$$\begin{array}{c} Q_{1}=\mu +\sigma_{p},\;Q_{2}=\mu +\sigma_{n},\;Q_{3}=\mu +\rho_{a},\;Q_{4}=\mu +\rho_{p},\;Q_{5}=\mu +\rho_{n}, \end{array}$$

$$\begin{array}{c} Q_{6}=\mu +\omega_{pp}+\omega_{pn},\;Q_{7}=\mu +\omega_{np}+\omega_{nn}.\; \end{array}$$

We first extract all downstream compartments in terms of the criminal stocks $C_{p}^{*}$ and $C_{n}^{*}$ by solving Eqs (12)–(19) in sequence.

From (16) we have


$$\begin{array}{c} J_{p}^{*}=\varphi_{1}\;C_{p}^{*},\;where\;\varphi_{1}=\frac{(1-\kappa )\sigma_{p}}{Q_{4}}. \end{array}$$ (20)

From (17) we have


$$\begin{array}{c} J_{n}^{*}=\varphi_{2}\;C_{n}^{*},\;where\;\varphi_{2}=\frac{\sigma_{n}}{Q_{5}}. \end{array}$$ (21)

From (15) we observe that


$$\begin{array}{c} A_{p}^{*}=\varphi_{3}\;C_{p}^{*},\;where\;\varphi_{3}=\frac{\kappa \sigma_{p}}{Q_{3}}. \end{array}$$ (22)

From (18), substituting $A_{p}^{*}$ and $J_{p}^{*}$ we have


$$\begin{array}{c} R_{p}^{*}=\varphi_{4}\;C_{p}^{*},\;where\;\varphi_{4}=\frac{\rho_{a}\;\varphi_{3}+(1-\pi )\rho_{p}\;\varphi_{1}}{Q_{6}}=\frac{1}{Q_{6}}\;\left[\frac{\kappa \sigma_{p}\rho_{a}}{Q_{3}}+\frac{(1-\kappa )\sigma_{p}(1-\pi )\rho_{p}}{Q_{4}}\right]. \end{array}$$ (23)

From (19), substituting the state variables $J_{n}^{*}$ and $J_{p}^{*}$ we have


$$\begin{array}{c} R_{n}^{*}=\varphi_{5}\;C_{n}^{*}+\varphi_{6}\;C_{p}^{*},\;where\;\varphi_{5}=\frac{\rho_{n}\;\varphi_{2}}{Q_{7}}=\frac{\rho_{n}\sigma_{n}}{Q_{5}Q_{7}},\;and\;\varphi_{6}=\frac{\pi \rho_{p}\;\varphi_{1}}{Q_{7}}=\frac{(1-\kappa )\sigma_{p}\pi \rho_{p}}{Q_{4}Q_{7}}. \end{array}$$ (24)

From (12) we have


$$\begin{array}{c} S^{*}=\frac{\Lambda }{\mu +\beta \;\left(C_{p}^{*}+\eta C_{n}^{*}\right)}. \end{array}$$ (25)

### Crime-free equilibrium

The crime-free equilibrium (CFE) is obtained by setting all crime-related compartments to zero and solving the resulting algebraic system. Equating the right-hand sides of (3)–(10) to zero and setting $C_{p}=C_{n}=A_{p}=J_{p}=J_{n}=R_{p}=R_{n}=0$ yields the unique CFE:


$$\begin{array}{c} {ℰ}_{0}=\left(\frac{\Lambda }{\mu },\;0,\;0,\;0,\;0,\;0,\;0,\;0\right). \end{array}$$

This equilibrium is biologically feasible and lies on the boundary of Ω.

## Crime generation number

### Geometric loop approach

To aid interpretation, we first describe the feedback loops that underlie the crime generation number ${ℛ}_{0}$. The crime generation number ${ℛ}_{0}$ quantifies the average number of secondary criminal cases produced by a single offender introduced into an otherwise crime-free, fully susceptible population. We derive ${ℛ}_{0}$ via a geometric series (transmission-loop) approach grounded in the next-generation matrix (NGM) framework of Diekmann et al. [34,35] and van den Driessche & Watmough [36]. Appendix B provides a formal proof that the spectral radius $\rho (ℱ{𝒱}^{-1})$ of the standard NGM coincides with the ${ℛ}_{0}$ derived below. The geometric approach is preferred here because it renders the contribution of each transmission loop explicitly and directly interpretable for policy analysis, a transparency not afforded by the aggregate spectral radius alone.

The force of crime initiation that is driven solely by $C_{p}$ and $C_{n}$ through λ. All downstream compartments ($A_{p},\;J_{p},\;J_{n},\;R_{p},\;R_{n}$) feed back into $C_{p}$ or $C_{n}$ via relapse or escalation pathways, but none of these compartments, including the community-supervision compartment $A_{p}$, appear in λ directly. The loops below trace the *indirect* routes by which diversion through $A_{p}$ can eventually return individuals to active offending. We identify and analyse these loops individually by considering the transitions of criminals as described in the model diagram:

**Petty non-custodial cycle:** $\left(C_{p}\to A_{p}\to R_{p}\to C_{p}\right)\text{.}$

A petty offender departs $C_{p}$ at rate $\kappa \sigma_{p}$ to receive a non-custodial sentence and enters the community-supervision stratum $A_{p}$, where they are removed from the ability to initiate new infections. Upon completing the supervised sentence, they transition to $R_{p}$ at rate $\rho_{a}$, and may subsequently relapse directly to petty crime $C_{p}$ at rate $\omega_{pp}$ (or escalate to serious crime $C_{n}$ at rate $\omega_{pn}$). The probability of completing this relapse loop, accounting for competing outflows at each stage, is


$$\begin{array}{c} \Phi_{1}^{\left(a\right)}=\left(\frac{\kappa \sigma_{p}}{Q_{1}}\right)\left(\frac{\rho_{a}}{Q_{3}}\right)\left(\frac{\omega_{pp}}{Q_{6}}\right). \end{array}$$ (26)

**Petty custodial, non-escalating branch:** $\left(C_{p}\to J_{p}\to R_{p}\to C_{p}\right)\text{.}$

A proportion (1−κ) of petty offenders enter custodial sentence $J_{p}$; of those released, a fraction (1−π) transition to $R_{p}$ at rate $\rho_{p}$ and subsequently relapse to petty crime $C_{p}$ at rate $\omega_{pp}$. The associated cycle factor is


$$\begin{array}{c} \Phi_{1}^{\left(b\right)}=\left(\frac{(1-\kappa )\sigma_{p}}{Q_{1}}\right)\left(\frac{(1-\pi )\rho_{p}}{Q_{4}}\right)\left(\frac{\omega_{pp}}{Q_{6}}\right). \end{array}$$ (27)

**Petty custodial, escalating then de-escalating:** $\left(C_{p}\to J_{p}\to R_{n}\to C_{p}\right)\text{.}$

The remaining fraction π of released $J_{p}$ individuals escalate to $R_{n}$ at rate $\rho_{p}$ but subsequently de-escalate back to petty crime $C_{p}$ at rate $\omega_{np}$. The cycle factor is


$$\begin{array}{c} \Phi_{1}^{\left(c\right)}=\left(\frac{(1-\kappa )\sigma_{p}}{Q_{1}}\right)\left(\frac{\pi \rho_{p}}{Q_{4}}\right)\left(\frac{\omega_{np}}{Q_{7}}\right). \end{array}$$ (28)

The total petty-crime internal cycle factor, aggregating all three loops, is


$$\begin{array}{c} \Phi_{1}=\Phi_{1}^{\left(a\right)}+\Phi_{1}^{\left(b\right)}+\Phi_{1}^{\left(c\right)}. \end{array}$$ (29)

For this to be well-defined as a probability, we require $\Phi_{1}<1$, which holds under the biological constraint that not all petty offenders cycle indefinitely. The mean total time a petty offender spends in $C_{p}$ across all cycles is then


$$\begin{array}{c} t_{C_{p}}^{in}=\frac{1}{Q_{1}(1-\Phi_{1})}. \end{array}$$

**Serious-crime custodial cycle:** $\left(C_{n}\to J_{n}\to R_{n}\to C_{n}\right)\text{.}$

A serious offender enters $J_{n}$ at rate $\sigma_{n}$, is released to $R_{n}$ at rate $\rho_{n}$, and relapses to $C_{n}$ at rate $\omega_{nn}$. The serious crime internal cycle factor is


$$\begin{array}{c} \Phi_{2}=\left(\frac{\sigma_{n}}{Q_{2}}\right)\left(\frac{\rho_{n}}{Q_{5}}\right)\left(\frac{\omega_{nn}}{Q_{7}}\right), \end{array}$$ (30)

with a mean total residence time in $C_{n}$ given by


$$\begin{array}{c} t_{C_{n}}^{in}=\frac{1}{Q_{2}(1-\Phi_{2})}. \end{array}$$

Cross-pathway escalation: ($C_{p}\to \{A_{p},\;R_{p},\;J_{p},\;R_{n}\}\to C_{n}$). Three distinct pathways allow a petty offender to escalate into the serious crime compartment $C_{n}$:


$$\begin{array}{ccc} \Phi_{pn}^{\left(a\right)} & = & \left(\frac{(1-\kappa )\sigma_{p}}{Q_{1}}\right)\left(\frac{\pi \rho_{p}}{Q_{4}}\right)\left(\frac{\omega_{nn}}{Q_{7}}\right), \end{array}$$ (31)

$$\begin{array}{ccc} \Phi_{pn}^{\left(b\right)} & = & \left(\frac{\kappa \sigma_{p}}{Q_{1}}\right)\left(\frac{\rho_{a}}{Q_{3}}\right)\left(\frac{\omega_{pn}}{Q_{6}}\right), \end{array}$$ (32)

$$\begin{array}{ccc} \Phi_{pn}^{\left(c\right)} & = & \left(\frac{(1-\kappa )\sigma_{p}}{Q_{1}}\right)\left(\frac{(1-\pi )\rho_{p}}{Q_{4}}\right)\left(\frac{\omega_{pn}}{Q_{6}}\right). \end{array}$$ (33)

Here, $\Phi_{pn}^{\left(a\right)}$ represents the carceral escalation pathway $C_{p}\to J_{p}\to R_{n}\to C_{n},$ a petty offender receives a custodial sentence, escalates upon release to $R_{n}$, and subsequently relapses into serious crime. $\Phi_{pn}^{\left(b\right)}$ represents escalation via the non-custodial route $C_{p}\to A_{p}\to R_{p}\to C_{n}$, and $\Phi_{pn}^{\left(c\right)}$ represents escalation via the non-escalating custodial route $C_{p}\to J_{p}\to R_{p}\to C_{n}$.

The composite cross-pathway escalation factor is


$$\begin{array}{c} \Phi_{pn}=\Phi_{pn}^{\left(a\right)}+\Phi_{pn}^{\left(b\right)}+\Phi_{pn}^{\left(c\right)}, \end{array}$$ (34)

and the mean time, a petty offender contributes to $C_{n}$ via these escalation pathways is $t_{C_{pn}}^{in}=\Phi_{pn}\;t_{C_{n}}^{in}$.

At the CFE ${ℰ}_{0}$, only the susceptible class $S_{0}=\Lambda /\mu$ is non-zero. The next-generation matrix $K=ℱ{𝒱}^{-1}$ has rank one (see Appendix B), so its spectral radius reduces to a scalar expression. Accounting for both the direct (primary) crime-generation pathways and the feedback loops, the crime generation number is


$$\begin{array}{c} {ℛ}_{0}=\underset{R_{0}^{c_{p}}}{\underbrace{\frac{q\beta \Lambda }{\mu \;Q_{1}(1-\Phi_{1})}}}+\underset{R_{0}^{c_{n}}}{\underbrace{\frac{\beta \eta \Lambda }{\mu \;Q_{2}(1-\Phi_{2})}\left[(1-q)+\frac{q\;\Phi_{pn}}{1-\Phi_{1}}\right]}}=R_{0}^{c_{p}}+R_{0}^{c_{n}}. \end{array}$$ (35)

The first component $R_{0}^{c_{p}}$ accounts for new petty-crime cases generated by a typical petty offender cycling through the petty-crime pathway. The second component $R_{0}^{c_{n}}$ accounts for new serious-crime cases arising from two sources: direct serious-crime recruitment (proportion 1−q), and petty offenders who escalate to serious crime via the composite cross-pathway $\Phi_{pn}$ (proportion *q*, weighted by the escalation factor).

**Remark:**

The carceral-induced criminality ratio (CCR), denoted $\Phi_{pn}^{\left(a\right)}$ and defined in (31), quantifies the probability that a custodially sentenced petty offender follows the escalation loop $J_{p}\to R_{n}\to C_{n}$, thereby generating serious crime. It is a product of three conditional probabilities: receiving a custodial sentence $\left(\frac{(1-\kappa )\sigma_{p}}{Q_{1}}\right)$, escalating to $R_{n}$ upon release $\left(\frac{\pi \rho_{p}}{Q_{4}}\right)$, and relapsing from $R_{n}$ into serious crime $\left(\frac{\omega_{nn}}{Q_{7}}\right)$. Unlike the composite $\Phi_{pn}$, which also responds to $\rho_{a}$ and additional pathways, $\Phi_{pn}^{\left(a\right)}$ is reducible by exactly two direct policy levers: increasing prison rehabilitation efficacy ($\rho_{p}$) or decreasing serious-offender relapse ($\omega_{nn}$). Thus, the CCR provides a targeted measure of the justice system’s capacity to inadvertently escalate petty offenders to serious criminals.

The community-supervision escalation ratio $\Psi_{pn}=(\frac{\kappa \sigma_{p}}{Q_{1}})(\frac{\rho_{a}}{Q_{3}})(\frac{\omega_{pn}}{Q_{6}})$ quantifies the probability that a petty offender diverted from custody progresses through the loop $A_{p}\to R_{p}\to C_{n}$ and escalates to serious crime. $\Psi_{pn}$ is a product of three policy-addressable probabilities; non-custodial sentencing, successful supervised rehabilitation, and cross-class escalation from $R_{p}$ to $C_{n}$.

The CCR ($\Phi_{pn}^{\left(a\right)}$) and CSER ($\Psi_{pn}$) represent the escalation risks of custodial versus non-custodial routes, respectively. They respond to different levers: $\omega_{pn}$ (post-diversion supervision) affects only $\Psi_{pn}$; $\rho_{p}$ (prison rehabilitation) affects only $\Phi_{pn}^{\left(a\right)}$; increasing κ (diversion rate) reduces $\Phi_{pn}^{\left(a\right)}$ but raises $\Psi_{pn}$, creating a policy tension measured by $\Phi_{pn}^{\left(a\right)}/\Psi_{pn}$. At baseline, $\Psi_{pn}\ll \Phi_{pn}^{\left(a\right)}$, confirming that custody carries greater escalation risk and supporting diversion policies provided that relapse prevention ($\omega_{pn}$ decreasing) is implemented concurrently.

#### Crime-persistent equilibrium.

To determine the crime-persistent equilibrium (CPE) we substitute the expressions (20)-(25) into the criminal-Eqs (13)–(14). Substituting

$R_{p}^{*}=\varphi_{4}C_{p}^{*}$ and $R_{n}^{*}=\varphi_{5}C_{n}^{*}+\varphi_{6}C_{p}^{*}$ into (13) we obtain


$$\begin{array}{c} q\beta (C_{p}^{*}+\eta C_{n}^{*})S^{*}=Q_{1}C_{p}^{*}-\omega_{pp}\varphi_{4}C_{p}^{*}-\omega_{np}\;\left(\varphi_{5}C_{n}^{*}+\varphi_{6}C_{p}^{*}\right). \end{array}$$

Defining the effective removal rate


$$\begin{array}{c} {\tilde{Q}}_{1}\;=\;Q_{1}-\omega_{pp}\varphi_{4}-\omega_{np}\varphi_{6}, \end{array}$$ (36)

this becomes


$$\begin{array}{c} q\beta (C_{p}^{*}+\eta C_{n}^{*})S^{*}={\tilde{Q}}_{1}\;C_{p}^{*}-\omega_{np}\varphi_{5}\;C_{n}^{*}. \end{array}$$ (36a)

Substituting the same expressions into (14) gives


$$\begin{array}{c} (1-q)\beta (C_{p}^{*}+\eta C_{n}^{*})S^{*}=Q_{2}C_{n}^{*}-\omega_{pn}\varphi_{4}C_{p}^{*}-\omega_{nn}\;\left(\varphi_{5}C_{n}^{*}+\varphi_{6}C_{p}^{*}\right). \end{array}$$

Defining


$$\begin{array}{c} {\tilde{Q}}_{2}\;=\;Q_{2}-\omega_{nn}\varphi_{5}, \end{array}$$ (37)

this becomes


$$\begin{array}{c} (1-q)\beta (C_{p}^{*}+\eta C_{n}^{*})S^{*}={\tilde{Q}}_{2}\;C_{n}^{*}-(\omega_{pn}\varphi_{4}+\omega_{nn}\varphi_{6})C_{p}^{*}. \end{array}$$ (37b)

Expanding $\varphi_{4}$ and $\varphi_{6}$ explicitly in (36), we factor *Q*_1_ from ${\tilde{Q}}_{1}$ so that


$$\begin{array}{c} {\tilde{Q}}_{1}=Q_{1}\;\left(1-\frac{\omega_{pp}\varphi_{4}+\omega_{np}\varphi_{6}}{Q_{1}}\right)\;\equiv \;Q_{1}(1-\Phi_{1}), \end{array}$$

where, substituting (22)–(24), yields


$$\begin{array}{c} \Phi_{1}=\Phi_{1}^{\left(a\right)}+\Phi_{1}^{\left(b\right)}+\Phi_{1}^{\left(c\right)}, \end{array}$$

where


$$\begin{array}{c} \Phi_{1}^{\left(a\right)}=\left(\frac{\kappa \sigma_{p}}{Q_{1}}\right)\left(\frac{\rho_{a}}{Q_{3}}\right)\left(\frac{\omega_{pp}}{Q_{6}}\right),\;\Phi_{1}^{\left(b\right)}=\left(\frac{(1-\kappa )\sigma_{p}}{Q_{1}}\right)\left(\frac{(1-\pi )\rho_{p}}{Q_{4}}\right)\left(\frac{\omega_{pp}}{Q_{6}}\right), \end{array}$$

$$\begin{array}{c} \Phi_{1}^{\left(c\right)}=\left(\frac{(1-\kappa )\sigma_{p}}{Q_{1}}\right)\left(\frac{\pi \rho_{p}}{Q_{4}}\right)\left(\frac{\omega_{np}}{Q_{7}}\right).\; \end{array}$$

Each term in the expression for $\Phi_{1}$ has a direct interpretation with regards to the movement of individuals. $\Phi_{1}^{\left(a\right)}$ is the $C_{p}\;\to \;A_{p}\;\to \;R_{p}\;\to \;C_{p}$ loop, $\Phi_{1}^{\left(b\right)}$ is the $C_{p}\;\to \;J_{p}\;\to \;R_{p}\;\to \;C_{p}$ loop (custodial, non-escalating fraction 1−π) and $\Phi_{1}^{\left(c\right)}$ is the $C_{p}\;\to \;J_{p}\;\to \;R_{n}\;\to \;C_{p}$ loop (custodial, escalating fraction π, relapse back to petty crime at rate $\omega_{np}$).

Similarly, factoring *Q*_2_ from ${\tilde{Q}}_{2}$ gives


$$\begin{array}{c} {\tilde{Q}}_{2}=Q_{2}(1-\Phi_{2}),\;where\;\Phi_{2}=\left(\frac{\sigma_{n}}{Q_{2}}\right)\left(\frac{\rho_{n}}{Q_{5}}\right)\left(\frac{\omega_{nn}}{Q_{7}}\right) \end{array}$$

corresponding to the single loop $C_{n}\;\to \;J_{n}\;\to \;R_{n}\;\to \;C_{n}$.

Dividing Eq (a) by *q* and (b) by (1−q) gives the same left-hand side $\beta (C_{p}^{*}+\eta C_{n}^{*})S^{*}$. Given that


$$\begin{array}{c} \frac{{\tilde{Q}}_{1}\;C_{p}^{*}-\omega_{np}\varphi_{5}\;C_{n}^{*}}{q}=\frac{{\tilde{Q}}_{2}\;C_{n}^{*}-(\omega_{pn}\varphi_{4}+\omega_{nn}\varphi_{6})C_{p}^{*}}{1-q}. \end{array}$$

we can solve for $C_{n}^{*}$ so that


$$\begin{array}{c} C_{n}^{*}=\Gamma \;C_{p}^{*}. \end{array}$$ (38)

where


$$\begin{array}{c} \Gamma \;=\;\frac{\left(1-q){\tilde{Q}}_{1}+q(\omega_{pn}\varphi_{4}+\omega_{nn}\varphi_{6}\right)}{(1-q)\omega_{np}\varphi_{5}+q{\tilde{Q}}_{2}}. \end{array}$$

Now substituting $C_{n}^{*}=\Gamma C_{p}^{*}$ and $S^{*}=\Lambda /(\mu +\beta (1+\eta \Gamma )C_{p}^{*})$ into Eq (a) yields


$$\begin{array}{c} \frac{q\beta \Lambda (1+\eta \Gamma )C_{p}^{*}}{\mu +\beta (1+\eta \Gamma )C_{p}^{*}}=({\tilde{Q}}_{1}-\omega_{np}\varphi_{5}\Gamma )C_{p}^{*}. \end{array}$$

We thus have $C_{p}^{*}=0$ corresponding to the crime-free equilibrium and the non-trivial case $C_{p}^{*}\neq 0$ gives the crime-persistent equilibrium. We note that


$$\begin{array}{c} C_{p}^{*}=\frac{\mu }{\beta (1+\eta \Gamma )}\left({ℛ}_{0}-1\right), \end{array}$$ (39)

where


$$\begin{array}{c} {ℛ}_{0}=\frac{q\beta \Lambda (1+\eta \Gamma )}{\mu \;\left({\tilde{Q}}_{1}-\omega_{np}\varphi_{5}\Gamma \right)}=\frac{q\beta \Lambda }{\mu Q_{1}(1-\Phi_{1})}+\frac{\beta \eta \Lambda }{\mu Q_{2}(1-\Phi_{2})}\left[(1-q)+\frac{q\;\Phi_{pn}}{1-\Phi_{1}}\right], \end{array}$$ (40)

and


$$\begin{array}{c} \Phi_{pn}=\Phi_{pn}^{\left(a\right)}+\Phi_{pn}^{\left(b\right)}+\Phi_{pn}^{\left(c\right)}, \end{array}$$

where


$$\begin{array}{c} \Phi_{pn}^{\left(a\right)}=\left(\frac{(1-\kappa )\sigma_{p}}{Q_{1}}\right)\left(\frac{\pi \rho_{p}}{Q_{4}}\right)\left(\frac{\omega_{nn}}{Q_{7}}\right),\;\Phi_{pn}^{\left(b\right)}=\left(\frac{\kappa \sigma_{p}}{Q_{1}}\right)\left(\frac{\rho_{a}}{Q_{3}}\right)\left(\frac{\omega_{pn}}{Q_{6}}\right), \end{array}$$

$$\begin{array}{c} \Phi_{pn}^{\left(c\right)}=\left(\frac{(1-\kappa )\sigma_{p}}{Q_{1}}\right)\left(\frac{(1-\pi )\rho_{p}}{Q_{4}}\right)\left(\frac{\omega_{pn}}{Q_{6}}\right).\; \end{array}$$

is the total cross-escalation probability from petty to serious crime.

A back substitution of the result in (39) into the remaining expressions gives the CPE.

## Stability of equilibria

### Stability of the crime-free equilibrium

Standard theory for compartmental models (van den Driessche & Watmough [36]) establishes the following result:

**Theorem 3.** *The CFE* ${ℰ}_{0}$ *is locally asymptotically stable when* ${ℛ}_{0}<1$ *and unstable whenever* ${ℛ}_{0}>1$*.*

### Existence and stability of the crime-persistent equilibrium

The crime-persistent equilibrium components are thus obtained by back-substitution of (39), so that


$$\begin{array}{cc} C_{n}^{*} & =\Gamma \;C_{p}^{*},\;A_{p}^{*}=\varphi_{3}\;C_{p}^{*},\;J_{p}^{*}=\varphi_{1}\;C_{p}^{*},\;J_{n}^{*}=\varphi_{2}\;C_{n}^{*},\;R_{p}^{*}=\varphi_{4}\;C_{p}^{*},\\ R_{n}^{*} & =\varphi_{5}\;C_{n}^{*}+\varphi_{6}\;C_{p}^{*},\;S^{*}=\frac{\Lambda }{\mu +\beta (C_{p}^{*}+\eta C_{n}^{*})}. \end{array}$$

We thus have the following result on the existence of the crime-persistent equilibrium.

**Theorem 4.**

*If* ${ℛ}_{0}>1$*, there exists a unique crime-persistent equilibrium*


$$\begin{array}{c} E_{1}=(S^{*},\;C_{p}^{*},\;C_{n}^{*},\;A_{p}^{*},\;J_{p}^{*},\;J_{n}^{*},\;R_{p}^{*},\;R_{n}^{*})\;\text{with all components strictly positive if }{ℛ}_{0}>1 \end{array}$$

*If* ${ℛ}_{0}\leq 1$*, the only non-negative equilibrium is E*_*0*_.

### Stability of the crime-persistent equilibrium

The stability of the crime-persistent equilibrium ${ℰ}_{1}$ is analysed via the block Jacobian matrix at ${ℰ}_{1}$. Partitioning the Jacobian into submatrices for susceptible, criminal, and rehabilitated classes simplifies the characteristic equation. Using Schur complement arguments to compute det(J−λI) isolates the eigenvalue spectrum and yields explicit stability conditions.

Consider the Jacobian matrix 𝒥 at the crime-persistent equilibrium ${ℰ}_{1}$. The variables are ordered as $\left(S,C_{p},C_{n},A_{p},J_{p},J_{n},R_{p},R_{n}\right)$. The Jacobian can be written in the following 4×4 block matrix form:


$$\begin{array}{c} 𝒥=\left[\begin{array}{cc} 𝒜 & ℬ\\ 𝒞 & 𝒟 \end{array}\right], \end{array}$$

where the blocks are defined by the partial derivatives of the system (3)–(10) evaluated at ${ℰ}_{1}$. The block matrices are thus given by


$$\begin{array}{c} 𝒜=\left(\begin{array}{cccc} -(\beta (C_{p}^{*}+\eta C_{n}^{*})+\mu ) & -\beta S^{*} & -\beta \eta S^{*} & 0\\ q\beta (C_{p}^{*}+\eta C_{n}^{*}) & q\beta S^{*}-Q_{1} & q\beta \eta S^{*} & 0\\ \left(1-q)\beta (C_{p}^{*}+\eta C_{n}^{*}\right) & (1-q)\beta S^{*} & (1-q)\beta \eta S^{*}-Q_{2} & 0\\ 0 & \kappa \sigma_{p} & 0 & -Q_{3} \end{array}\right), \end{array}$$

$$\begin{array}{c} ℬ=\left(\begin{array}{cccc} 0 & 0 & 0 & 0\\ 0 & 0 & \omega_{pp} & \omega_{np}\\ 0 & 0 & \omega_{pn} & \omega_{nn}\\ 0 & 0 & 0 & 0 \end{array}\right),\;𝒞=\left(\begin{array}{cccc} 0 & (1-\kappa )\sigma_{p} & 0 & 0\\ 0 & 0 & \sigma_{n} & 0\\ 0 & 0 & 0 & \rho_{a}\\ 0 & 0 & 0 & 0 \end{array}\right), \end{array}$$

$$\begin{array}{c} 𝒟=\left(\begin{array}{cccc} -Q_{4} & 0 & 0 & 0\\ 0 & -Q_{5} & 0 & 0\\ (1-\pi )\rho_{p} & 0 & -Q_{6} & 0\\ \pi \rho_{p} & \rho_{n} & 0 & -Q_{7} \end{array}\right). \end{array}$$

The characteristic polynomial is given by $\det(𝒥-\lambda I_{8})=0$. Using the Schur complement formula for block matrices, given that $𝒟-\lambda I_{4}$ is invertible, we have:


$$\begin{array}{c} \det(𝒥-\lambda I_{8})=\det(𝒟-\lambda I_{4})\cdot \det\left(𝒜-\lambda I_{4}-ℬ(𝒟-\lambda I_{4}{)}^{-1}𝒞\right). \end{array}$$

Hence, the eigenvalues of 𝒥 are obtained from:


$$\begin{array}{cc} \det(𝒟-\lambda I_{4}) & =0,\\ \det\left(𝒜-\lambda I_{4}-ℬ(𝒟-\lambda I_{4}{)}^{-1}𝒞\right) & =0. \end{array}$$ (41)

Given that the block matrix 𝒟 is a lower triangular matrix, the eigenvalues obtained from $\det(𝒟-\lambda I_{4})=0$ are simply its diagonal entries:


$$\begin{array}{c} \lambda_{i}=\{-Q_{4},-Q_{5},-Q_{6},-Q_{7}\},\;\text{for }i=5,6,7,8. \end{array}$$

These eigenvalues are clearly negative since $Q_{4},Q_{5},Q_{6},Q_{7}>0.$

Considering the correction term $ℬ(𝒟-\lambda I_{4}{)}^{-1}𝒞$, we note that ℬ and 𝒞 are sparse; only a few entries of the correction term are nonzero. First, we compute $(𝒟-\lambda I_{4}{)}^{-1}$. Since $𝒟-\lambda I_{4}$ is lower triangular, its inverse is also lower triangular and multiplying $(𝒟-\lambda I_{4}{)}^{-1}$ by 𝒞, then left-multiplying by ℬ, yields a sparse correction matrix $ℰ(\lambda )=ℬ(𝒟-\lambda I_{4}{)}^{-1}𝒞$. After some tedious algebraic manipulations, we obtain the following nonzero entries:


$$\begin{array}{c} {ℰ}_{2,2}(\lambda )=\frac{\omega_{pp}(1-\pi )\rho_{p}(1-\kappa )\sigma_{p}}{\left(Q_{4}+\lambda )(Q_{6}+\lambda \right)}+\frac{\omega_{np}\pi \rho_{p}(1-\kappa )\sigma_{p}}{\left(Q_{4}+\lambda )(Q_{7}+\lambda \right)}+\frac{\omega_{np}(1-\pi )\rho_{p}\rho_{n}(1-\kappa )\sigma_{p}}{\left(Q_{4}+\lambda )(Q_{6}+\lambda )(Q_{7}+\lambda \right)}, \end{array}$$

$$\begin{array}{c} {ℰ}_{2,3}(\lambda )=\frac{\omega_{np}\rho_{n}\sigma_{n}}{\left(Q_{5}+\lambda )(Q_{7}+\lambda \right)},\;{ℰ}_{2,4}(\lambda )=\frac{\omega_{pp}\rho_{a}}{Q_{6}+\lambda }, \end{array}$$

$$\begin{array}{c} {ℰ}_{3,2}(\lambda )=\frac{\omega_{pn}(1-\pi )\rho_{p}(1-\kappa )\sigma_{p}}{\left(Q_{4}+\lambda )(Q_{6}+\lambda \right)}+\frac{\omega_{nn}\pi \rho_{p}(1-\kappa )\sigma_{p}}{\left(Q_{4}+\lambda )(Q_{7}+\lambda \right)}+\frac{\omega_{nn}(1-\pi )\rho_{p}\rho_{n}(1-\kappa )\sigma_{p}}{\left(Q_{4}+\lambda )(Q_{6}+\lambda )(Q_{7}+\lambda \right)}, \end{array}$$

$$\begin{array}{c} {ℰ}_{3,3}(\lambda )=\frac{\omega_{nn}\rho_{n}\sigma_{n}}{\left(Q_{5}+\lambda )(Q_{7}+\lambda \right)},\;{ℰ}_{3,4}(\lambda )=\frac{\omega_{pn}\rho_{a}}{Q_{6}+\lambda }. \end{array}$$

We now define the 4×4 matrix $ℳ(\lambda )=𝒜-\lambda I_{4}-ℰ(\lambda )$. Substituting the expressions above results in the following


$$\begin{array}{c} ℳ(\lambda )=\left(\begin{array}{cccc} J_{SS}^{*}-\lambda & -\beta S^{*} & -\beta \eta S^{*} & 0\\ q\beta (C_{p}^{*}+\eta C_{n}^{*}) & q\beta S^{*}-Q_{1}-\lambda -{ℰ}_{2,2}(\lambda ) & q\beta \eta S^{*}-{ℰ}_{2,3}(\lambda ) & -{ℰ}_{2,4}(\lambda )\\ \left(1-q)\beta (C_{p}^{*}+\eta C_{n}^{*}\right) & \left(1-q)\beta S^{*}-{ℰ}_{3,2}(\lambda \right) & J_{nn}^{*} & -{ℰ}_{3,4}(\lambda )\\ 0 & \kappa \sigma_{p} & 0 & -Q_{3}-\lambda \end{array}\right), \end{array}$$

where $J_{SS}^{*}=-(\beta (C_{p}^{*}+\eta C_{n}^{*})+\mu )$ and $J_{nn}^{*}=(1-q)\beta \eta S^{*}-Q_{2}-\lambda -{ℰ}_{3,3}(\lambda ).$

To compute det(ℳ(λ)), we can perform a cofactor expansion along the fourth row, which has the form:


$$\begin{array}{c} \det(ℳ(\lambda ))=\kappa \sigma_{p}\;\det({ℳ}_{4,2}(\lambda ))+(-Q_{3}-\lambda )\;\det({ℳ}_{4,4}(\lambda )), \end{array}$$

where ${ℳ}_{4,2}(\lambda )$ is the minor obtained by deleting the fourth row and second column, and ${ℳ}_{4,4}(\lambda )=\tilde{ℳ}$ is the minor obtained by deleting the fourth row and fourth column.


$$\begin{array}{c} \tilde{ℳ}=\left(\begin{array}{ccc} J_{SS}^{*}-\lambda & -\beta S^{*} & -\beta \eta S^{*}\\ q\beta (C_{p}^{*}+\eta C_{n}^{*}) & q\beta S^{*}-Q_{1}-\lambda -{ℰ}_{2,2}(\lambda ) & q\beta \eta S^{*}-{ℰ}_{2,3}(\lambda )\\ \left(1-q)\beta (C_{p}^{*}+\eta C_{n}^{*}\right) & \left(1-q)\beta S^{*}-{ℰ}_{3,2}(\lambda \right) & \left(1-q)\beta \eta S^{*}-Q_{2}-\lambda -{ℰ}_{3,3}(\lambda \right) \end{array}\right). \end{array}$$

The minor ${ℳ}_{4,2}(\lambda )$ is the 3×3 matrix obtained by deleting the fourth row and second column:


$$\begin{array}{c} {ℳ}_{4,2}(\lambda )=\left(\begin{array}{ccc} J_{SS}^{*}-\lambda & -\beta \eta S^{*} & 0\\ q\beta (C_{p}^{*}+\eta C_{n}^{*}) & q\beta \eta S^{*}-{ℰ}_{2,3}(\lambda ) & -{ℰ}_{2,4}(\lambda )\\ \left(1-q)\beta (C_{p}^{*}+\eta C_{n}^{*}\right) & \left(1-q)\beta \eta S^{*}-Q_{2}-\lambda -{ℰ}_{3,3}(\lambda \right) & -{ℰ}_{3,4}(\lambda ) \end{array}\right). \end{array}$$

Therefore, the characteristic equation for the remaining eigenvalues is given by


$$\begin{array}{c} \det(ℳ(\lambda ))=-\kappa \sigma_{p}\det({ℳ}_{4,2}(\lambda ))+(-Q_{3}-\lambda )\det({ℳ}_{4,4}(\lambda ))=0. \end{array}$$ (42)

Eq (42) is a transcendental equation in λ due to the rational functions ${ℰ}_{i,j}(\lambda )$ appearing in the minors.

Local asymptotic stability of ${ℰ}_{1}$ is assessed by linearising system (3)–(10) about ${ℰ}_{1}$. The Jacobian $J({ℰ}_{1})$ yields a characteristic polynomial, and the Routh–Hurwitz conditions for local asymptotic stability require that all coefficients of the characteristic polynomial be positive and that all relevant Hurwitz determinants be positive. Denoting the characteristic polynomial of the reduced 3×3 Jacobian block $\tilde{ℳ}$ as $p(\lambda )=\lambda^{3}+a_{1}\lambda^{2}+a_{2}\lambda +a_{3}$, the three Routh–Hurwitz conditions are:

(i) *a*_1_ > 0, (ii) *a*_3_ > 0, and (iii) $a_{1}a_{2}>a_{3}$.

These conditions have been verified numerically over a Latin Hypercube sample of 1,000 parameter vectors drawn from the ranges in Table 2; across all samples satisfying ${ℛ}_{0}>1$, all three conditions hold. The eigenvalues of $J({ℰ}_{1})$ at the baseline parameterisation are reported in Table 1, and all have strictly negative real parts, confirming local asymptotic stability.

**Table 1:** Eigenvalues of the Jacobian at the baseline CPE ${ℰ}_{1}$.

Eigenvalue	Approximate value
$\lambda_{1}=-Q_{4}$	−0.307
$\lambda_{2}=-Q_{5}$	−0.114
$\lambda_{3}=-Q_{6}$	−0.214
$\lambda_{4}=-Q_{7}$	−0.364
$\lambda_{5}=-Q_{3}$	−0.614
$\lambda_{6,7,8}$	Computed numerically; all Re(λ)<0

**Table 2:** Model parameters, baseline values, sources, and type.

Param.	Value	Source
Λ	320,000 yr^-1^	2% growth, Gauteng 16M [38]
β	$3.5\times 10^{-8}$	Calibrated to SAPS [1]
η	2	Gang influence [24,25]
*q*	0.75	SAPS petty crime share [1]
κ	0.40	JICS non-custodial rate [30]
$\sigma_{p}$	0.5 yr^-1^	SALRC petty processing [40]
$\sigma_{n}$	0.33 yr^-1^	SALRC serious processing [40]
$\rho_{a}$	0.6 yr^-1^	DCS/Muntingh [28,29]
$\rho_{p}$	0.3 yr^-1^	DCS [29]
$\rho_{n}$	0.1 yr^-1^	Singh [39]
$\omega_{p}$	0.2 yr^-1^	Samuels et al. [32]
$\omega_{n}$	0.35 yr^-1^	Lötter [33]
μ	0.014 yr^-1^	World Bank [41]

**Proposition 1.** *If* ${ℛ}_{0}>1$*, the unique CPE* ${ℰ}_{1}$ *is locally asymptotically stable for the given baseline parameter set and across the parameter ranges in* Table 1*, as confirmed by the Routh-Hurwitz conditions and numerical eigenvalue analysis [*37*].*

## Numerical simulations

To investigate the impact of sentencing and rehabilitation policies on long-run crime dynamics in South Africa, we perform numerical simulations of system (3)–(10) using parameter values derived from the available literature and official statistical reports. The model is implemented in MATLAB using a standard fourth-order Runge–Kutta scheme and integrated over a 30-year horizon. The simulations are initialised using demographic and criminal justice data for Gauteng province, whose total population was approximately 16 million in 2023.

### Parameter estimation

The recruitment rate Λ=320,000 yr^-1^ follows from a 2% annual growth rate for Gauteng’s 16 million population [38]. The contact rate $\beta =3.5\times 10^{-8}$ (criminal susceptible)${\;}^{-1}\text{yr}{\;}^{-1}$ is calibrated to yield an equilibrium petty-crime prevalence $C_{p}^{*}/N^{*}\approx 0.60$, consistent with SAPS data [1]. The non-custodial rehabilitation rate $\rho_{a}=0.6$ yr^-1^ is based on DCS community corrections [29] and Muntingh [28]. Custodial rehabilitation rates $\rho_{p}=0.3$ yr^-1^ and $\rho_{n}=0.1$ yr^-1^ reflect lower prison efficacy, consistent with DCS statistics [29] and Singh [39]. Aggregate relapse rates $\omega_{p}=0.2$ yr^-1^ and $\omega_{n}=0.35$ yr^-1^ come directly from South African studies [32,33]. For parameters drawn from international literature (e.g., the model architecture and peer-influence factor η), the qualitative dynamics are structurally robust across contexts [12,16], and the sensitivity analysis confirms stability of conclusions over the full plausible parameter range.

A complete table of model parameters, their baseline values, and sources is given in Table 2, see also Appendix A.

The model is initialised using 2023 Gauteng demographic and criminal justice data from the Department of Correctional Services, with the assumption that Gauteng accounts for more than 25% of South Africa’s total prison population [42,43]. The initial conditions are set as follows:


$$\begin{array}{c} S(0)=15,000,000;\;C_{p}(0)=200,000;\;C_{n}(0)=50,000;\;J_{p}(0)=25,000; \end{array}$$

$$\begin{array}{c} J_{n}(0)=15,000;\;A_{p}(0)=30,000;\;R_{p}(0)=20,000;\;R_{n}(0)=10,000. \end{array}$$

## Global sensitivity analysis

**Fig 2:**
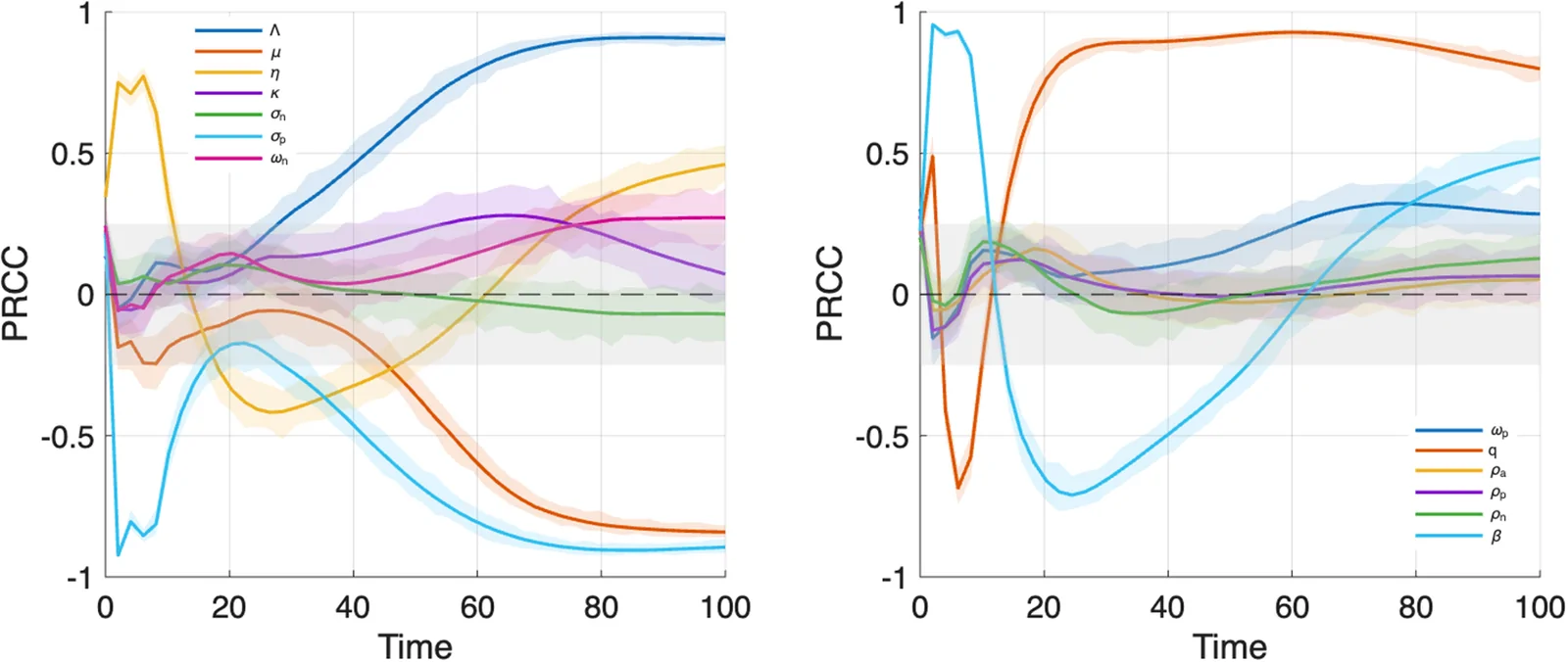
PRCC profiles over time for $C_{p}(t)$: (a) parameters $\Lambda ,\mu ,q,\omega_{p},\sigma_{p}$; (b) parameters $\kappa ,\rho_{a},\rho_{p},\rho_{n},\beta ,\eta$. Shaded bands represent 95% confidence intervals.

**Fig 3:**
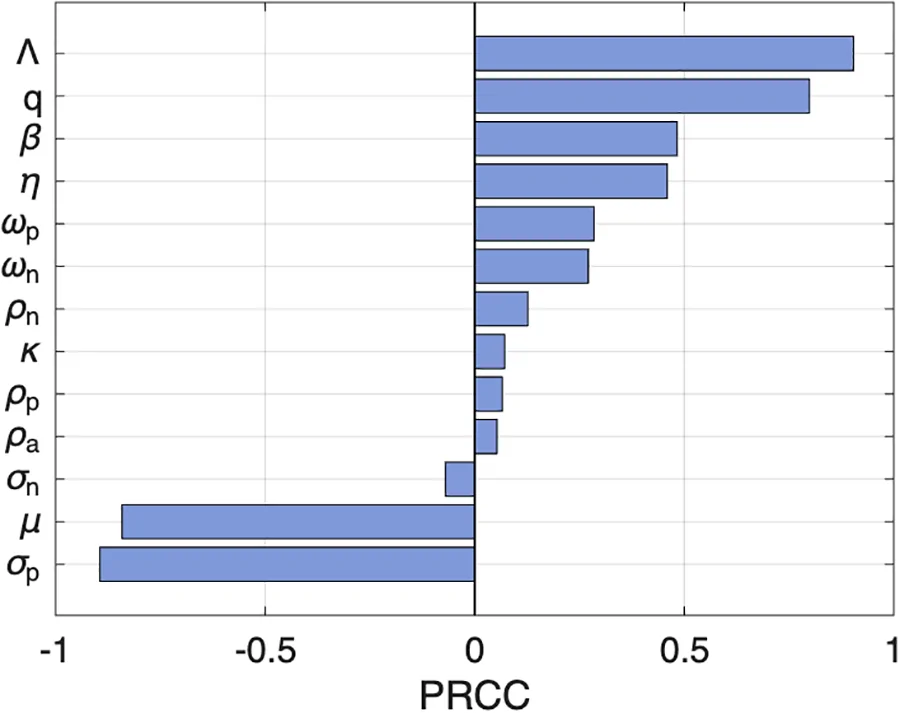
PRCC profiles over time for $C_{n}(t)$: (a) and (b) as in Fig 2. (a) Tornado plot of final-time PRCCs for $C_{p}(t)$. (b) Tornado plot of final-time PRCCs for $C_{n}(t)$.

**Fig 4:**
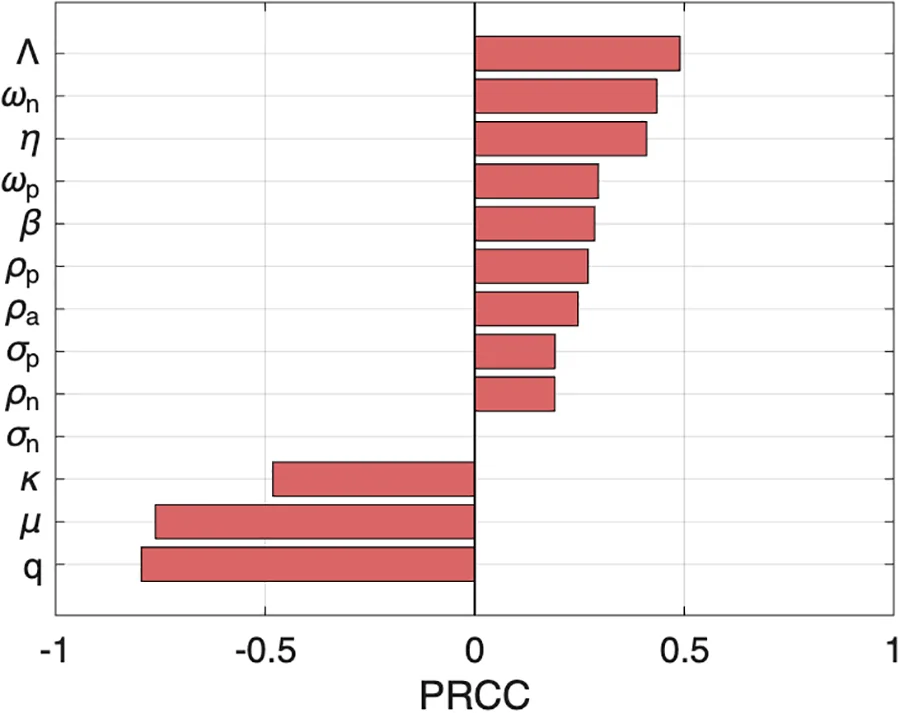
Partial rank correlation coefficient (PRCC) tornado plots at the final simulation time identify the most influential parameters for crime prevalence. Positive PRCC values (rightward bars) indicate a direct (increasing) effect, negative values (leftward bars) an inverse (reducing) effect. For petty crime $C_{p}(t)$, the strongest positive drivers are κ (non-custodial sentencing proportion) and Λ (recruitment rate), while μ (mortality) and $\sigma_{p}$ (conviction rate) exert the strongest reducing influence. For serious crime $C_{n}(t)$, the dominant positive drivers are Λ, $\omega_{nn}$ (serious-crime relapse rate), and $\sigma_{n}$ (serious-crime conviction rate). Bar length ranks each parameter by absolute PRCC magnitude.

Global sensitivity analysis (GSA) employed Latin Hypercube Sampling (LHS) with Partial Rank Correlation Coefficients (PRCC) [44,45]. Five hundred parameter sets were drawn from the ranges in Table 2, and the model was simulated over a 100-year horizon per set. Time-varying PRCC values were computed for the crime compartments $C_{p}(t)$ and $C_{n}(t)$; PRCC ranks inputs and outputs, measures correlation while controlling for other parameters, and captures monotonic (including nonlinear) relationships [45]. Bootstrap confidence intervals (100 resamples per time point) assessed statistical robustness; shaded regions denote 95% confidence bands, and a grey band between −0.25 and 0.25 indicates negligible influence. Figs 2–4 present the resulting PRCC profiles. Tornado plots at the final simulation time rank parameters by the magnitude of their PRCC values; positive values indicate direct effects, negative values inverse relationships, and the magnitude reflects relative importance.

To enhance policy relevance, parameters are classified as either direct policy targets (modifiable by legislation or administration) or systemic outcomes (emergent from broader institutional processes). The non-custodial sentencing proportion κ and the non-custodial rehabilitation rate $\rho_{a}$ are direct policy targets with strong PRCC effects and immediate feasibility. In contrast, the petty-crime conviction rate $\sigma_{p}$ is systemic, alterable only through longer-term institutional change. The newly introduced CSER ($\Psi_{pn}$) identifies $\omega_{pn}$ as an additional direct policy target: it governs escalation from the petty rehabilitated class $R_{p}$ to serious crime $C_{n}$, a risk pathway activated by successful diversion through $A_{p}$. Reducing $\omega_{pn}$ via structured post-diversion supervision complements prison-rehabilitation strategies that target the CCR.

The PRCC analysis identifies Λ and *q* as the strongest positive drivers of petty crime $C_{p}(t)$, while μ and $\sigma_{p}$ act as reducers, see Table 3. The parameter κ exhibits the largest positive PRCC on $C_{p}(t)$, and $\rho_{a}$, $\rho_{p}$, $\rho_{n}$, β, and η are moderate curbing factors. For serious crime $C_{n}(t)$: Λ, $\omega_{nn}$, and $\sigma_{n}$ are the dominant positive drivers, while μ and $\rho_{a}$ act to reduce serious crime. Fig 2 displays the time-varying PRCC profiles for $C_{p}(t)$, and Fig 3 displays the corresponding profiles for $C_{n}(t)$.

**Table 3:** Parameter classification by policy actionability and direction of PRCC on $C_{p}(t)$, including CSER-relevant parameters.

Parameter	Type	PRCC on $C_{p}$	Policy lever	Metric affected
κ	Direct target	+ (large)	Sentencing reform	CCR-decreases CSER-increases
$\rho_{a}$	Direct target	- (moderate)	Diversion programmes	CSER-decreases
$\rho_{p}$	Direct target	- (moderate)	Prison rehabilitation	CCR-decreases
$\omega_{pn}$	Direct target	+ (moderate)	Post-diversion supervision	CSER-decreases
$\omega_{nn}$	Direct target	+ (moderate)	Relapse prevention	CCR-decreases
$\sigma_{p}$	Systemic	–	Policing and court efficiency	Both
$\omega_{p}$	Systemic	+ (small)	Reintegration processes	CSER
Λ	Systemic	+ (large)	Social policy environment	Both
*q*	Systemic	+ (large)	Indirect structural drivers	Both

## Model calibration and projection

### Model fitting to data

The model was calibrated against annual South African prison population data spanning 1995–2024 (Table 4). Parameter estimation was carried out by minimising the sum of squared residuals between model predictions for the total incarcerated population $J_{p}(t)+J_{n}(t)$ and the observed data, using a nonlinear least-squares routine in MATLAB.

**Table 4:** Interpolated South African Prison Population by Year. Sources: [42,43].

Year	Prison Pop.	Year	Prison Pop.
1995	117,487	2010	158,165
1996	115,254	2011	156,407
1997	125,704	2012	154,648
1998	142,045	2013	158,316
1999	146,590	2014	161,984
2000	178,998	2015	163,057
2001	183,319	2016	164,129
2002	187,640	2017	159,283
2003	168,971	2018	154,437
2004	150,302	2019	148,830
2005	158,071	2020	143,223
2006	165,840	2021	150,140
2007	164,576	2022	157,056
2008	163,312	2023	156,828
2009	160,739	2024	156,600

**Fig 5:**
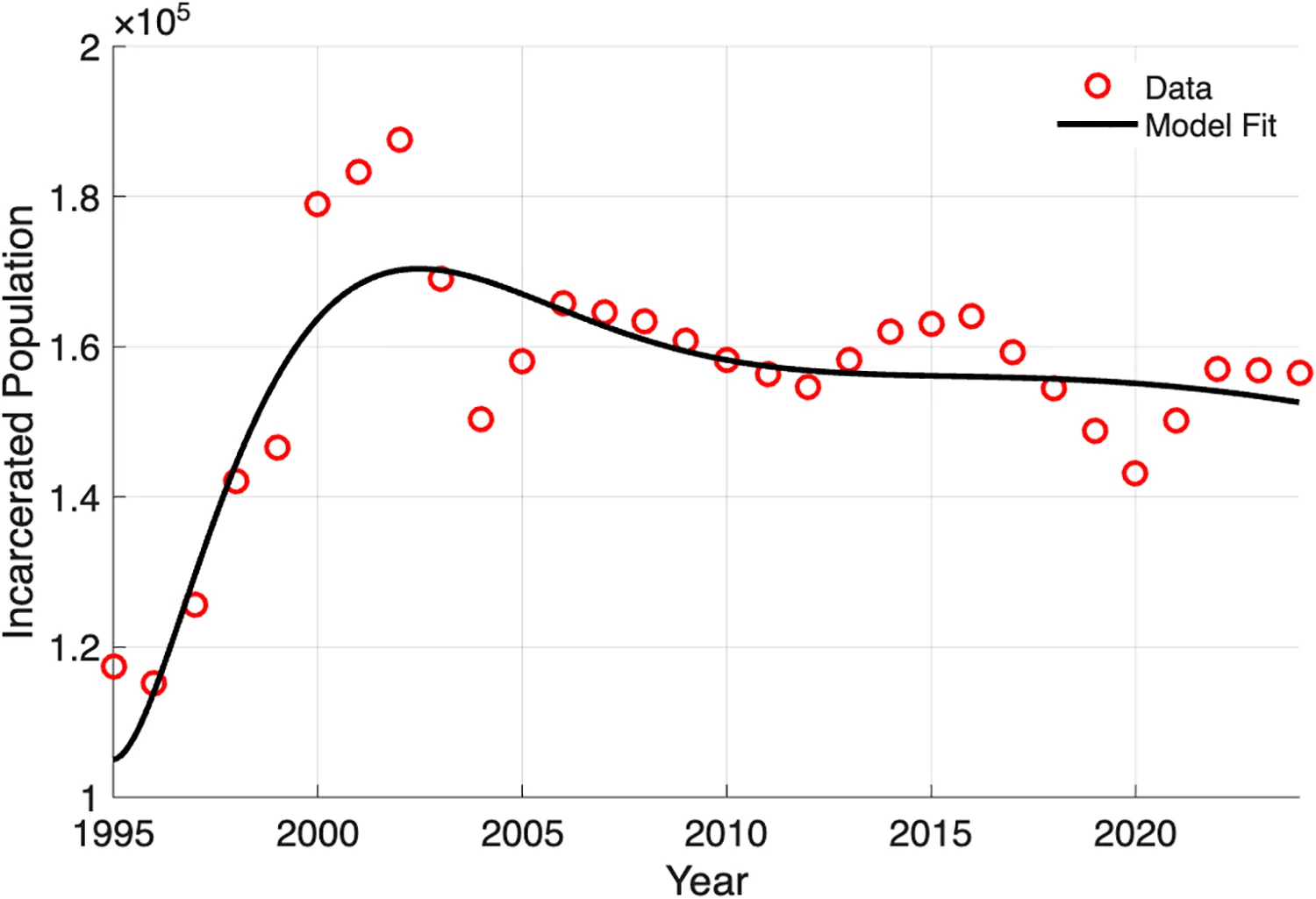
Best-fit model calibration to observed South African incarcerated population data, 1995–2024. Solid line: model prediction; circles: observed data. Horizontal axis shows calendar year, with *t* = 0 corresponding to 1995. Fitted parameters: $\beta =8.3455\times 10^{-5}$, η=1.1146, *q* = 0.0035229, κ=0.95389, $\sigma_{p}=0.15487$, $\sigma_{n}=1.7137\times 10^{-5}$, $\omega_{p}=0.84767$, $\omega_{n}=2.2986$, $\rho_{a}=0.012031$, $\rho_{p}=0.0066303$, $\rho_{n}=0.072207$, μ=0.019155, Λ=72,277.

Fig 5 presents the calibration of the model to South African incarceration data. The model underestimates the sharp peak observed near 1999–2002, when the prison population reached approximately 187,640. This discrepancy is attributable to two concurrent policy shocks: (i) the Criminal Procedure Second Amendment Act (Act 85 of 1997), which introduced mandatory minimum sentences effective from 1998, precipitating an abrupt increase in custodial sentences [46]; and (ii) post-apartheid policing reforms that substantially increased arrest and conviction rates between 1998 and 2002 [2]. These discrete, non-smooth policy shocks cannot be fully reproduced by the continuous ODE framework without incorporating time-varying parameters, a recognised limitation of the present model.

**Fig 6:**
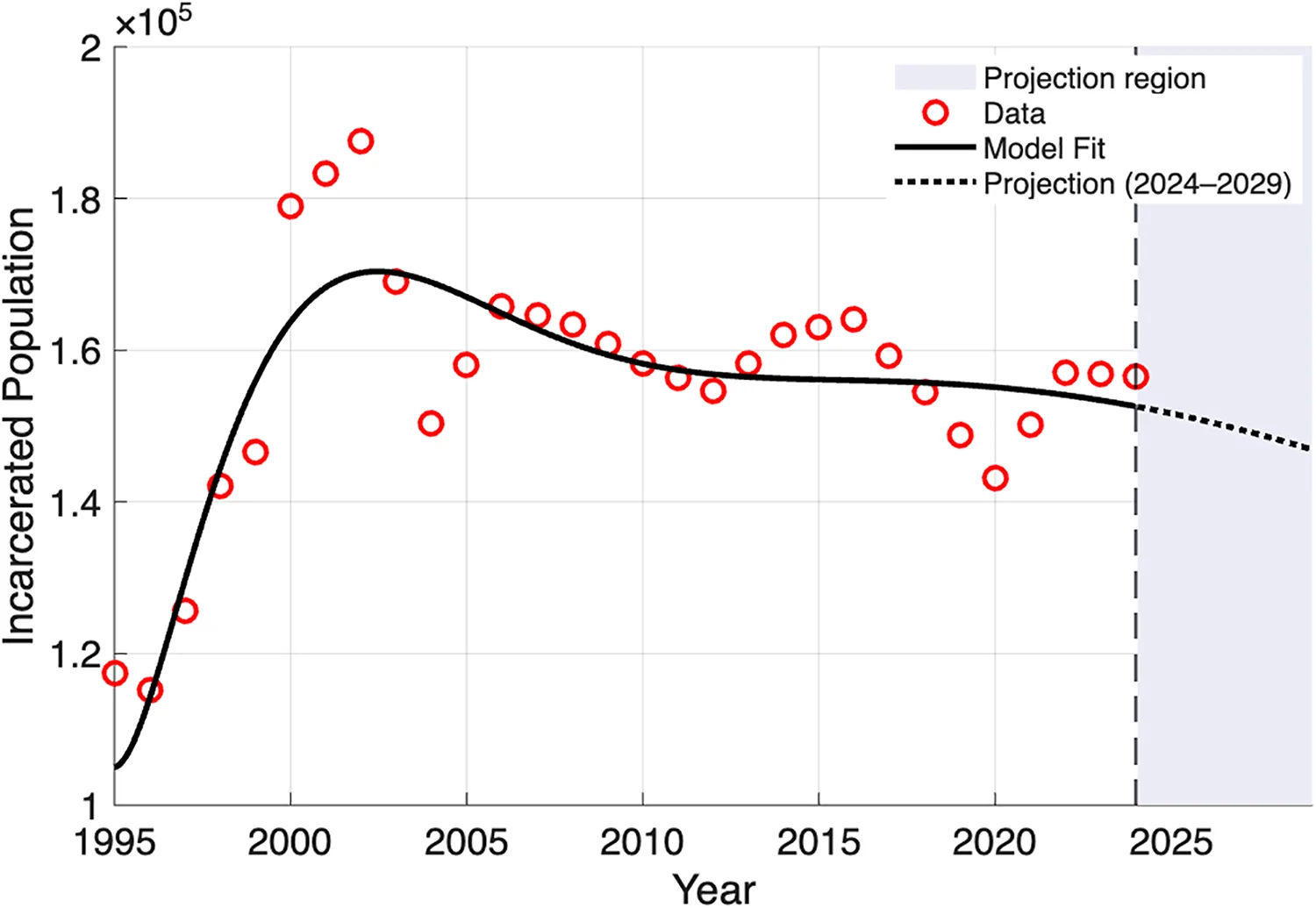
Best-fit model with a 5-year projection, 1995–2029. Solid line: model fit to observed data; dotted line: out-of-sample projection for 2024–2029, demarcated by a vertical dashed line. Under continuation of the 2024 fitted-parameter regime, the total incarcerated population is projected to decline gradually, consistent with the long-run attractor dynamics of the crime-persistent equilibrium ${ℰ}_{1}$ at the estimated ${ℛ}_{0}\approx 1.21$.

Fig 6 presents the best-fit model alongside a five-year out-of-sample projection through 2029.

**Fig 7:**
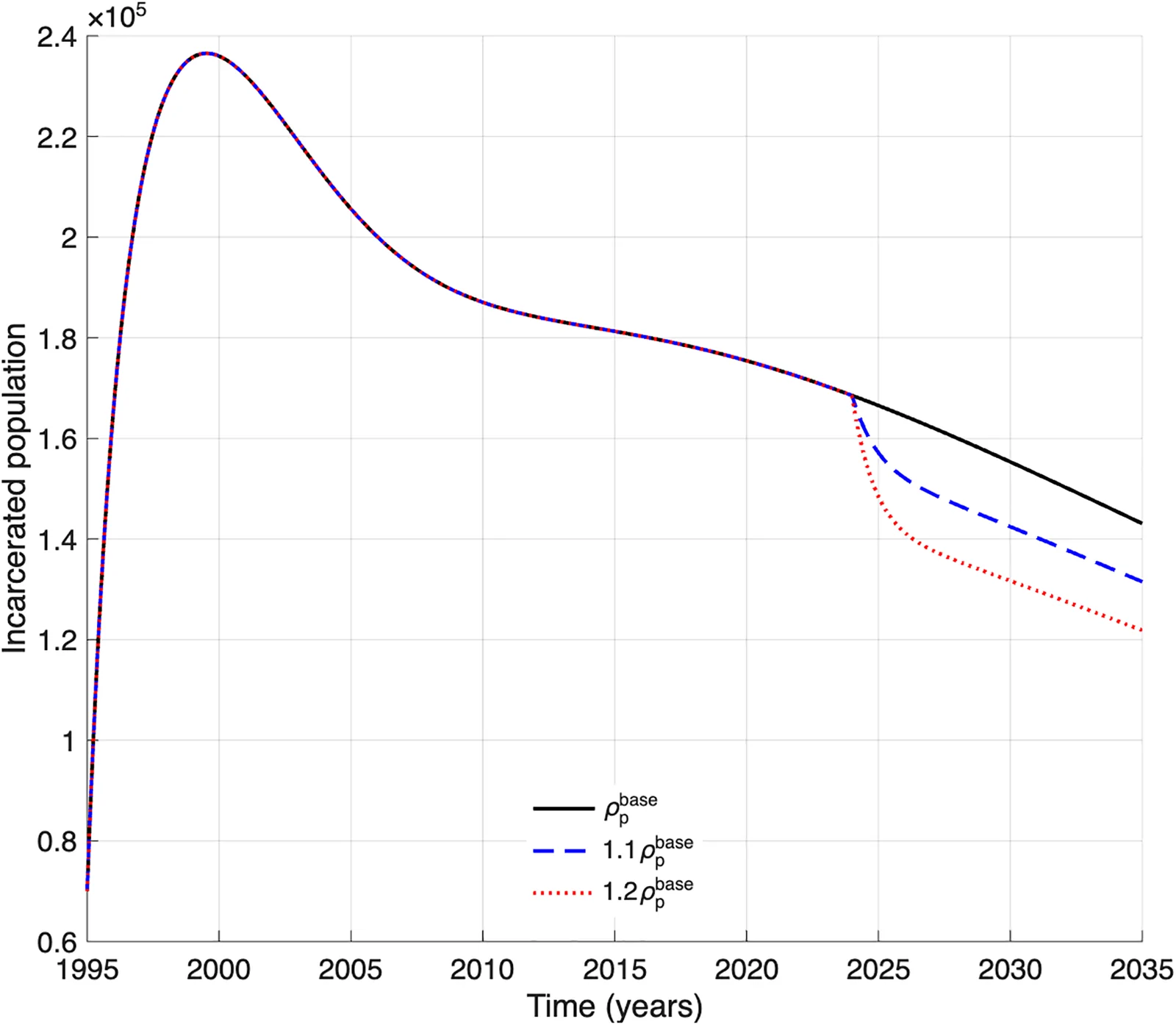
Impact of improving the custodial rehabilitation rate $\rho_{p}$ on the total incarcerated population $J_{p}(t)+J_{n}(t)$, 1995–2035. Horizontal axis: calendar year. Baseline: $\rho_{p}=0.0120$ yr^−1^ (solid black line; data up to 2024, thereafter model projection); dashed and dotted lines show scenarios with $\rho_{p}$ increased by factors of 1.1 and 1.2, respectively. Even modest improvements in prison rehabilitation efficacy generate a sustained downward trajectory in the incarcerated population over the simulation period, illustrating the cumulative long-run benefit of investment in custodial rehabilitation programmes.

**Fig 8:**
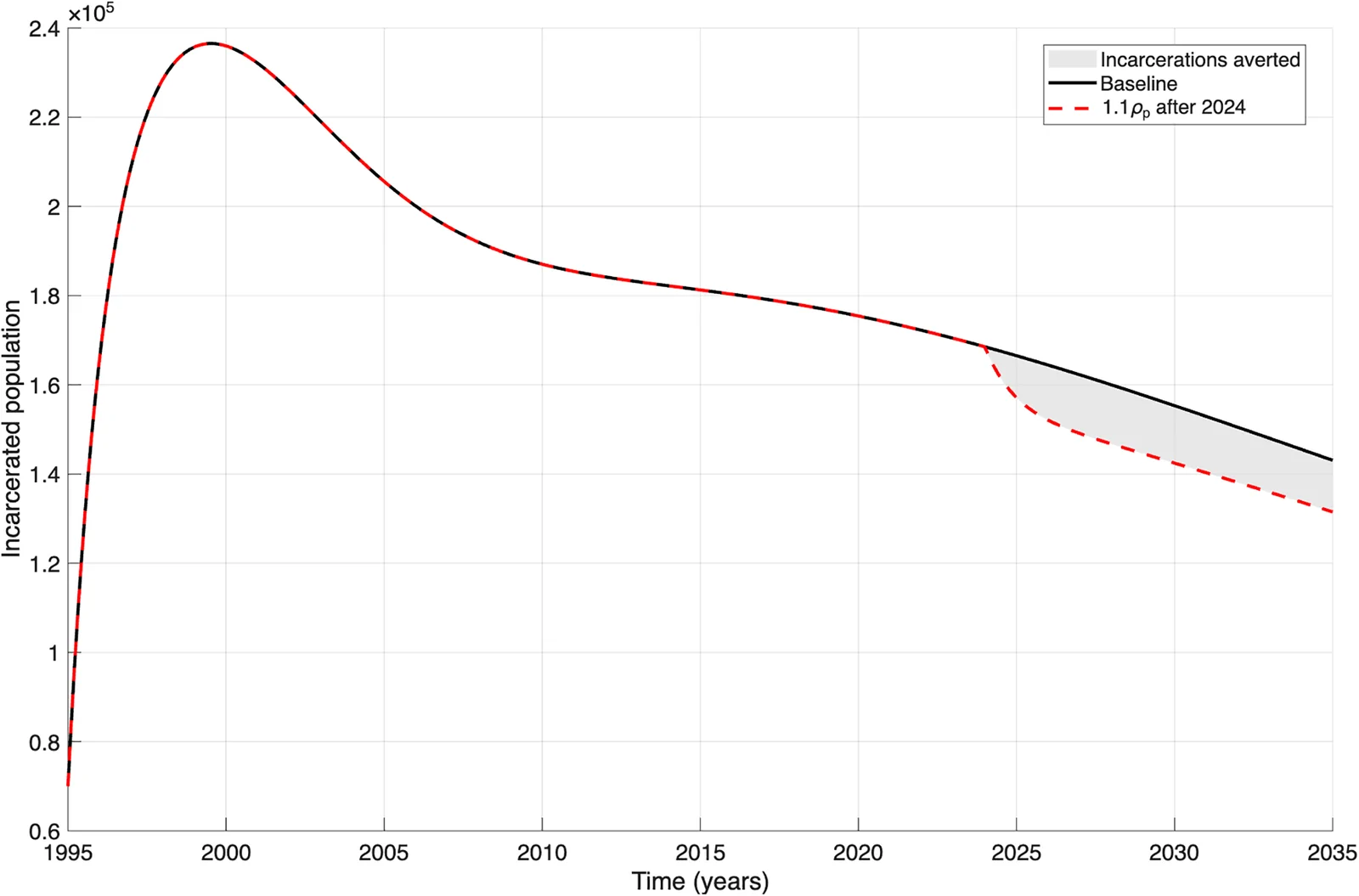
Projected incarcerated population $J_{p}(t)+J_{n}(t)$ under a policy-enhanced rehabilitation scenario, 1995–2035. Blue solid line: baseline trajectory with $\rho_{p}=0.012$ yr^-1^ (calibrated value); red dashed line: improved scenario with $\rho_{p}=0.018$ yr^-1^ (a 50% increase above baseline), intervention effective from ≈2012. The grey-shaded region quantifies cumulative incarcerations averted; its widening over time illustrates the compounding nature of rehabilitation gains.

Fig 7 illustrates the impact of improving the custodial rehabilitation rate $\rho_{p}$ on the total incarcerated population over the simulation time. Fig 8 shows projected incarceration trajectories under a policy-enhanced rehabilitation scenario, with the grey-shaded region quantifying the cumulative incarcerations averted.

### Relationship between incarceration and crime rates

The model makes the link between incarceration and crime explicit. At equilibrium, the incarcerated populations are proportional to the petty- and serious-crime rates,


$$\begin{array}{c} J_{p}^{*}=\frac{(1-\kappa )\sigma_{p}}{\mu +\rho_{p}}\;C_{p}^{*},\;J_{n}^{*}=\frac{\sigma_{n}}{\mu +\rho_{n}}\;C_{n}^{*}, \end{array}$$

so total incarceration is a severity-weighted reflection of active offending, with the proportionality determined by the custodial-sentencing fraction (1−κ), conviction rates $\sigma_{p},\sigma_{n}$, and release/rehabilitation rates $\rho_{p},\rho_{n}$. Away from equilibrium, $J_{p}$ and $J_{n}$ act as lagged accumulations of convictions and releases, producing characteristic delays of approximately $1/(\mu +\rho_{p})$ and $1/(\mu +\rho_{n})$, respectively. This explains why incarceration trajectories follow underlying crime dynamics with a delay. Increasing the custodial rehabilitation rate $\rho_{p}$ reduces incarceration both directly, by decreasing the petty-custody stock, and indirectly, by weakening the carceral escalation pathway $J_{p}\;\to \;R_{n}\;\to \;C_{n}$ (captured by the CCR, $\Phi_{pn}^{\left(a\right)}$), thereby lowering serious crime and the associated serious-custody population. As a result, reductions in incarceration reflect simultaneous reductions in imprisonment and serious-crime prevalence rather than merely shifting offenders out of custody. The cumulative incarcerations averted therefore provide a conservative indicator of the broader crime-reduction benefit and can be linked to the corresponding petty- and serious-crime trajectories.

### Asymmetric cross-pathway escalation

**Fig 9:**
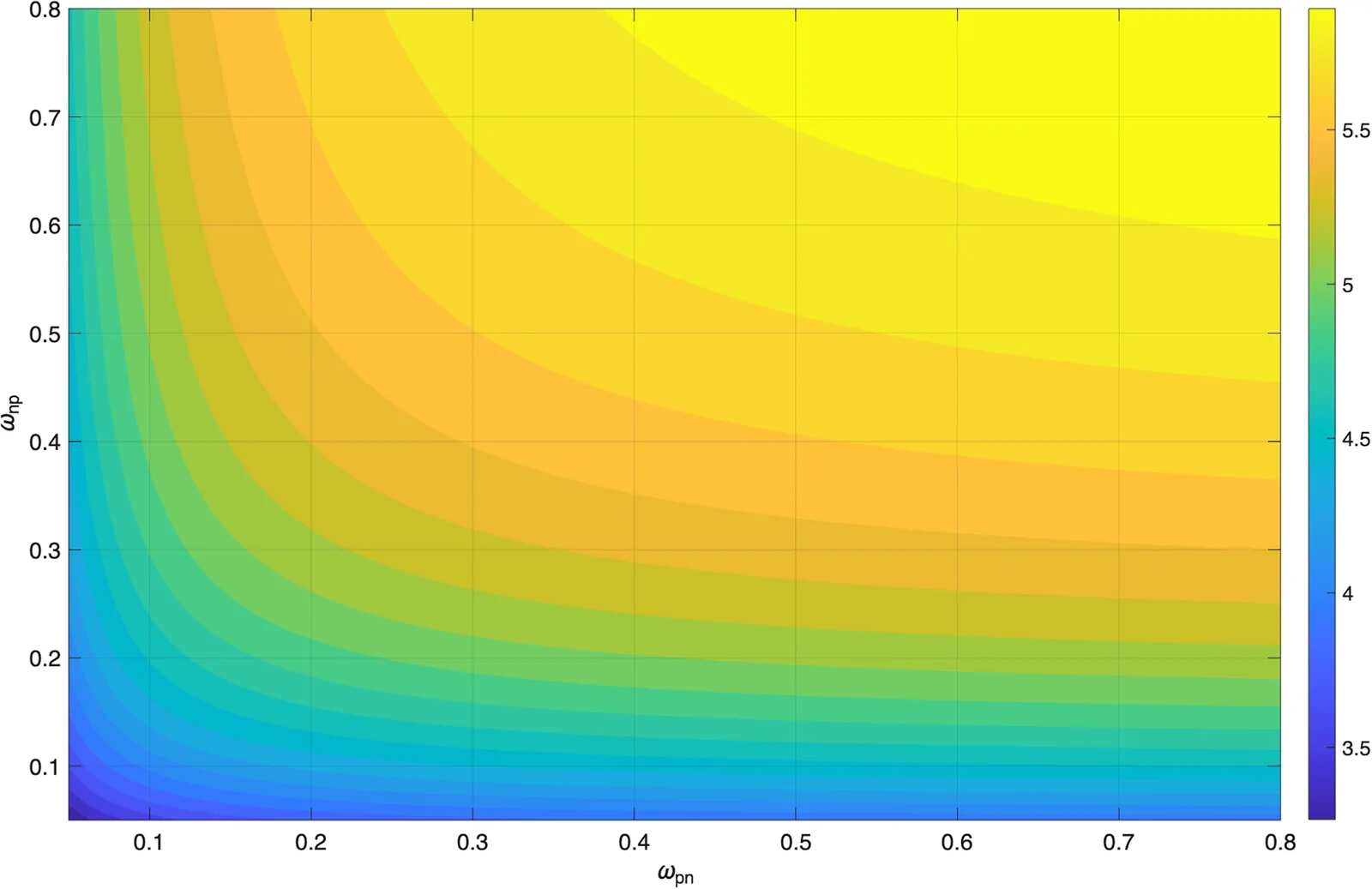
Joint sensitivity of the composite cross-pathway escalation factor $\Phi_{pn}$ to the escalation rate $\omega_{pn}$ (horizontal axis) and the de-escalation rate $\omega_{np}$ (vertical axis) at the baseline parameter set (Table 2). Colour intensity encodes $\Phi_{pn}$; warmer hues indicate higher escalation risk. The surface is strongly asymmetric: $\Phi_{pn}$ rises sharply with $\omega_{pn}$ but is nearly insensitive to $\omega_{np}$, confirming the empirically documented escalation-dominant regime [32,33]. The horizontal gradient far exceeds the vertical gradient, implying that policies reducing $\omega_{pn}$ (strengthening relapse prevention for petty rehabilitated offenders) yield substantially greater reductions in $\Phi_{pn}$ than equivalent efforts to lower de-escalation barriers from $R_{n}$.

A central feature of the relapse dynamics is the asymmetry between upward escalation from the petty rehabilitated class $R_{p}$ to serious crime $C_{n}$ (rate $\omega_{pn}$) and downward de-escalation from the serious rehabilitated class $R_{n}$ back to petty crime $C_{p}$ (rate $\omega_{np}$). This asymmetry is structurally embedded in the composite cross-pathway escalation factor


$$\begin{array}{c} \Phi_{pn}=\Phi_{pn}^{\left(a\right)}+\Phi_{pn}^{\left(b\right)}+\Phi_{pn}^{\left(c\right)}, \end{array}$$

which aggregates all routes by which petty offenders feed into serious-crime dynamics. Fig 9 explores this asymmetry over the full parameter domain of $\left(\omega_{pn},\omega_{np}\right)$. The figure further shows that the community-supervision escalation ratio (CSER, $\Psi_{pn}$) is highly sensitive to $\omega_{pn}$: even modest increases in $\omega_{pn}$ markedly expand the region where ${ℛ}_{0}>1$, whereas de-escalation ($\omega_{np}$) provides only weak attenuation. This asymmetry implies that effective post-diversion supervision must target $\omega_{pn}$ to contain the residual escalation risk of non-custodial sentencing.

### Influence of sentencing and rehabilitation parameters on $\Phi_{pn}^{\left(a\right)}$

This section examines the dependence of the carceral-induced criminality ratio $\Phi_{pn}^{\left(a\right)}$ on key sentencing, rehabilitation, and relapse parameters. Recall that $\Phi_{pn}^{\left(a\right)}$, defined in Eq (31), measures the probability that a single custodially sentenced petty offender traverses the full escalation loop $J_{p}\to R_{n}\to C_{n}$ and thereby generates serious criminal activity. Its three constituent probabilities, the probability of receiving a custodial sentence, the probability of escalating to $R_{n}$ upon release, and the probability of relapsing into serious crime from $R_{n}$, each admit direct policy intervention.

**Fig 10:**
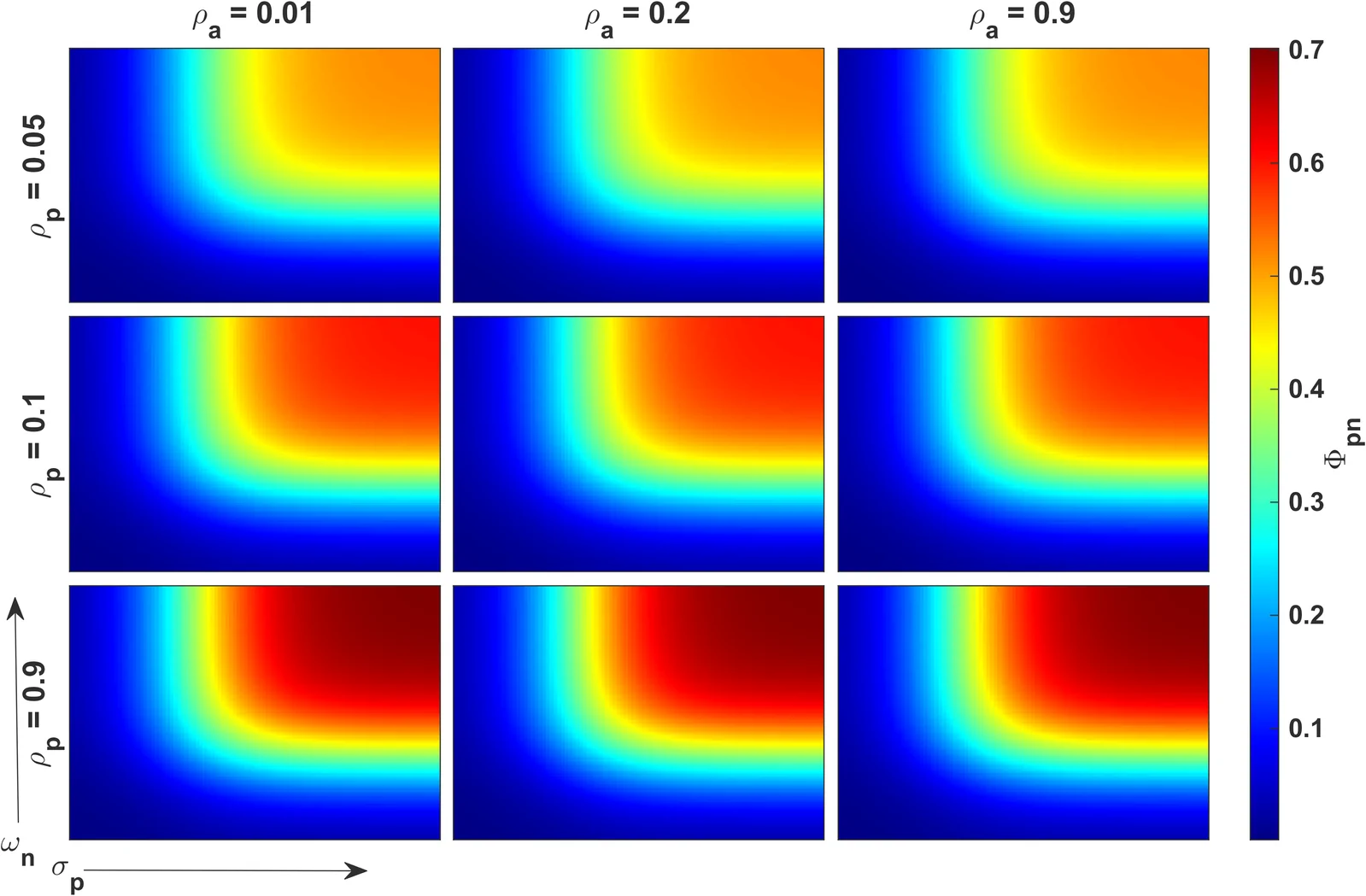
Heatmaps of the carceral-induced criminality ratio $\Phi_{pn}^{\left(a\right)}$ as a joint function of the petty-crime conviction rate $\sigma_{p}$ (horizontal axis) and the serious-crime relapse rate $\omega_{n}$ (vertical axis), both over [0.05,0.80] yr^-1^ on a 100×100 grid. The 3×3 subplots correspond to three levels of the custodial rehabilitation rate $\rho_{p}$ (rows, increasing downward) and three levels of the non-custodial rehabilitation rate $\rho_{a}$ (columns, increasing rightward). Three patterns emerge: (i) $\Phi_{pn}^{\left(a\right)}$ increases monotonically with both $\sigma_{p}$ and $\omega_{n}$; (ii) increasing $\rho_{p}$ suppresses $\Phi_{pn}^{\left(a\right)}$; (iii) counter-intuitively, increasing $\rho_{a}$ can elevate $\Phi_{pn}^{\left(a\right)}$ by enlarging the $R_{p}$ pool and amplifying relapse flux into $C_{n}$.

Fig 10 reveals that an increase in $\rho_{a}$ can paradoxically elevate $\Phi_{pn}^{\left(a\right)}$, whereas an increase in $\rho_{p}$ consistently reduces it. Increases in $\sigma_{p}$ and $\omega_{nn}$ both amplify $\Phi_{pn}^{\left(a\right)}$. The analytical expression


$$\begin{array}{c} \Phi_{pn}^{\left(a\right)}=\left(\frac{(1-\kappa )\sigma_{p}}{Q_{1}}\right)\left(\frac{\pi \rho_{p}}{Q_{4}}\right)\left(\frac{\omega_{nn}}{Q_{7}}\right) \end{array}$$

does not depend directly on $\rho_{a}$. However, dynamically, a higher $\rho_{a}$ increases throughput through $A_{p}\to R_{p}$, amplifying the relapse flux $\omega_{pp}R_{p}+\omega_{pn}R_{p}$ back into $C_{p}$ and $C_{n}$. The enlarged $C_{p}$ pool elevates the flow through the carceral escalation pathway $C_{p}\to J_{p}\to R_{n}$, thereby raising $\Phi_{pn}^{\left(a\right)}$ dynamically when relapse prevention is inadequate. The policy implication is clear: expanding non-custodial diversion ($\rho_{a}$ increasing) without simultaneously strengthening relapse prevention ($\omega_{pn}$ decreasing, $\omega_{pp}$ decreasing) may inadvertently sustain, or even amplify, the carceral escalation cycle.

### Impact of rehabilitation and relapse rates on ${ℛ}_{0}$

**Fig 11:**
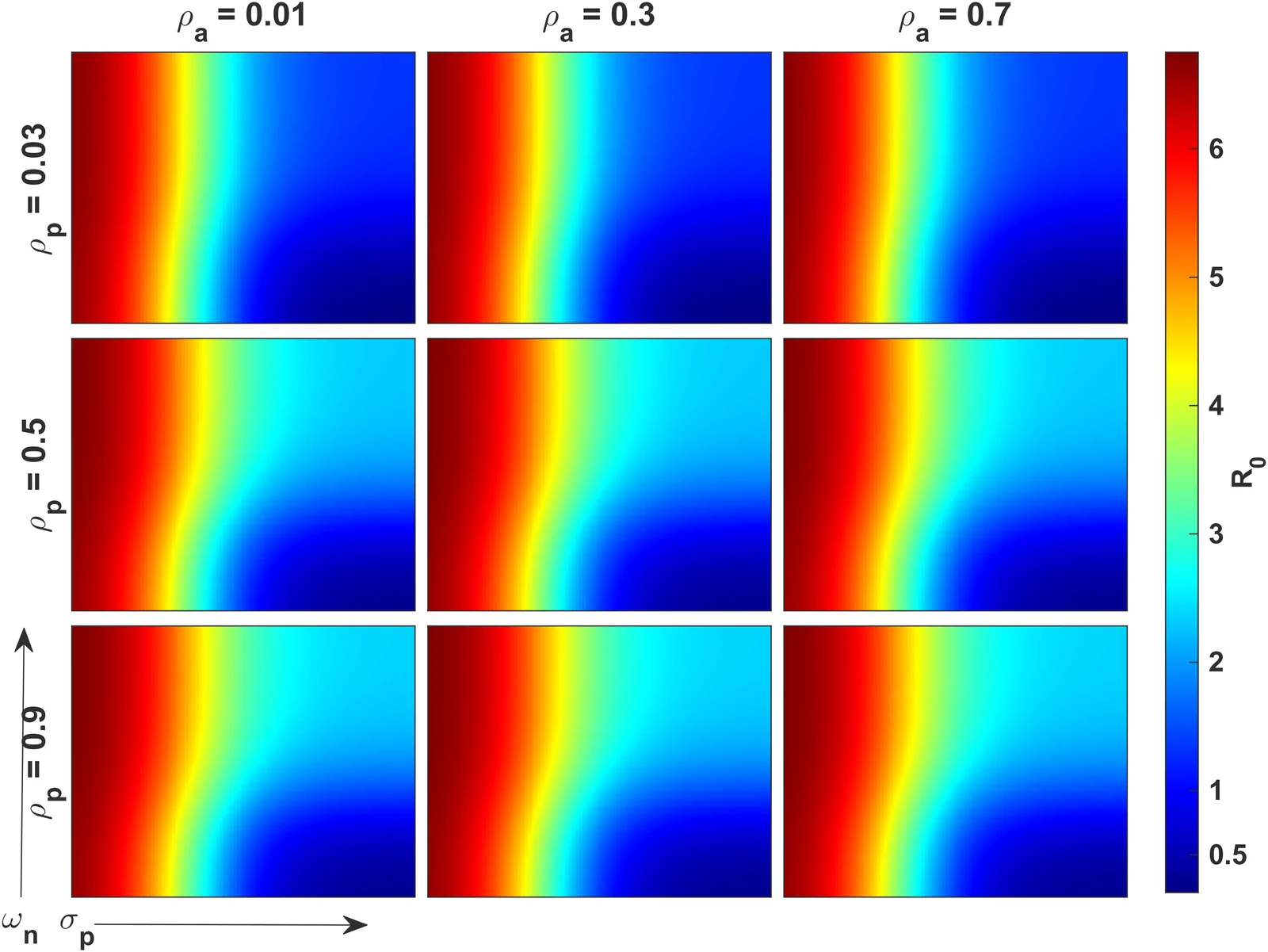
Heatmaps of the crime generation number ${ℛ}_{0}$ as a joint function of the petty-crime conviction rate $\sigma_{p}$ (horizontal) and the serious-crime relapse rate $\omega_{n}$ (vertical), each on [0.05,0.80] yr^-1^ over a 100×100 grid. The nine subplots vary $\rho_{p}$ (rows, increasing downward) and $\rho_{a}$ (columns, increasing rightward). Four main findings: (1) increasing $\sigma_{p}$ reduces ${ℛ}_{0}$; (2) increasing $\omega_{n}$ raises ${ℛ}_{0}$; (3) increasing $\rho_{p}$ (custodial rehabilitation) strongly compresses ${ℛ}_{0}$ toward elimination; (4) increasing $\rho_{a}$ can paradoxically raise ${ℛ}_{0}$ when $\omega_{pn}>0$ by enlarging the petty-rehabilitated pool and amplifying cross-class escalation.

**Fig 12:**
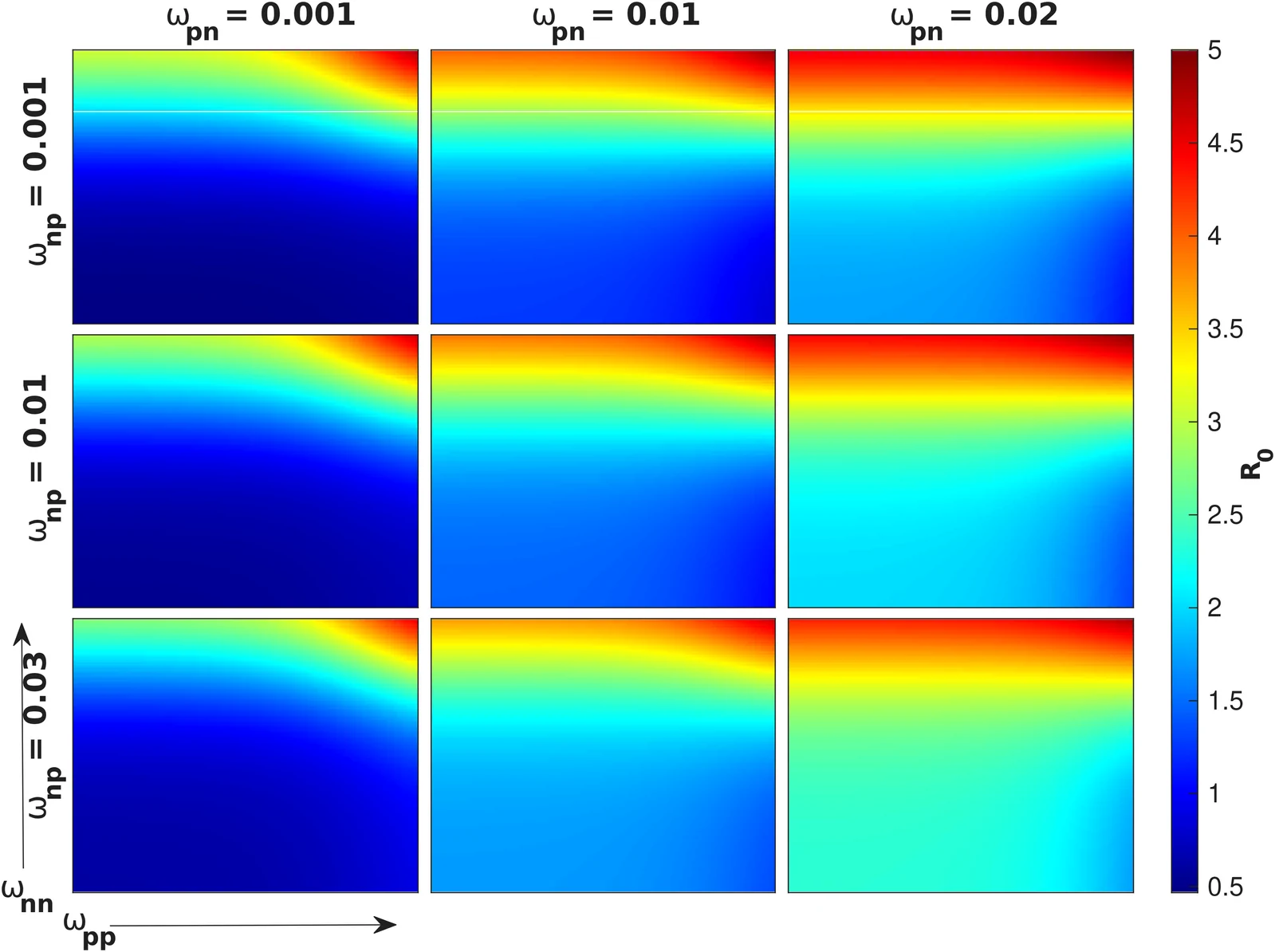
Heatmaps of ${ℛ}_{0}$ as a joint function of own-class relapse rates $\omega_{pp}$ (horizontal axis) and $\omega_{nn}$ (vertical axis), across a 3×3 grid of cross-class parameters: escalation rate $\omega_{pn}$ (columns, increasing rightward) and de-escalation rate $\omega_{np}$ (rows, increasing downward). Within each subplot, ${ℛ}_{0}$ increases with both $\omega_{pp}$ and $\omega_{nn}$, confirming that reducing own-class relapse suppresses crime generation. Across subplots, increasing $\omega_{pn}$ shifts the ${ℛ}_{0}=1$ boundary rightward, enlarging the crime-persistence region, whereas varying $\omega_{np}$ has a modest effect. Thus, cross-pathway escalation ($\omega_{pn}$) is a disproportionately influential driver of ${ℛ}_{0}$, and policies that reduce it offer the highest leverage for reducing aggregate crime propagation.

We now examine the sensitivity of the crime generation number ${ℛ}_{0}$ to the full set of rehabilitation and relapse parameters. Figs 11 and 12 present complementary heatmap analyses: Fig 11 varies the structural throughput parameters $\sigma_{p}$ and $\omega_{nn}$ across levels of $\rho_{p}$ and $\rho_{a}$, while Fig 12 varies the four pathway-specific relapse rates $\omega_{pp}$, $\omega_{nn}$, $\omega_{pn}$, and $\omega_{np}$.

Fig 11 reveals a delicate parameter balance: increasing $\sigma_{p}$ reduces ${ℛ}_{0}$; increasing $\omega_{nn}$ modestly raises it; increasing $\rho_{p}$ consistently lowers ${ℛ}_{0}$; and increasing $\rho_{a}$ can raise ${ℛ}_{0}$ in the absence of adequate relapse prevention, consistent with the mechanism identified for $\Phi_{pn}^{\left(a\right)}$ above.

At the baseline parameterisation (Table 2), ${ℛ}_{0}\approx 1.21$, indicating a regime of sustained but moderate crime propagation in which each offender generates approximately 1.21 new offenders in a fully susceptible population. Doubling the non-custodial sentencing proportion κ from 0.40 to 0.80, representing a major legislative shift, reduces ${ℛ}_{0}$ to approximately 0.87 < 1, implying that crime would decline toward the CFE. At Gauteng’s population scale, this corresponds to averting approximately 6,200 new petty-crime cases per year, providing a concrete quantitative illustration of the public safety benefit of sentencing reform.

**Fig 13:**
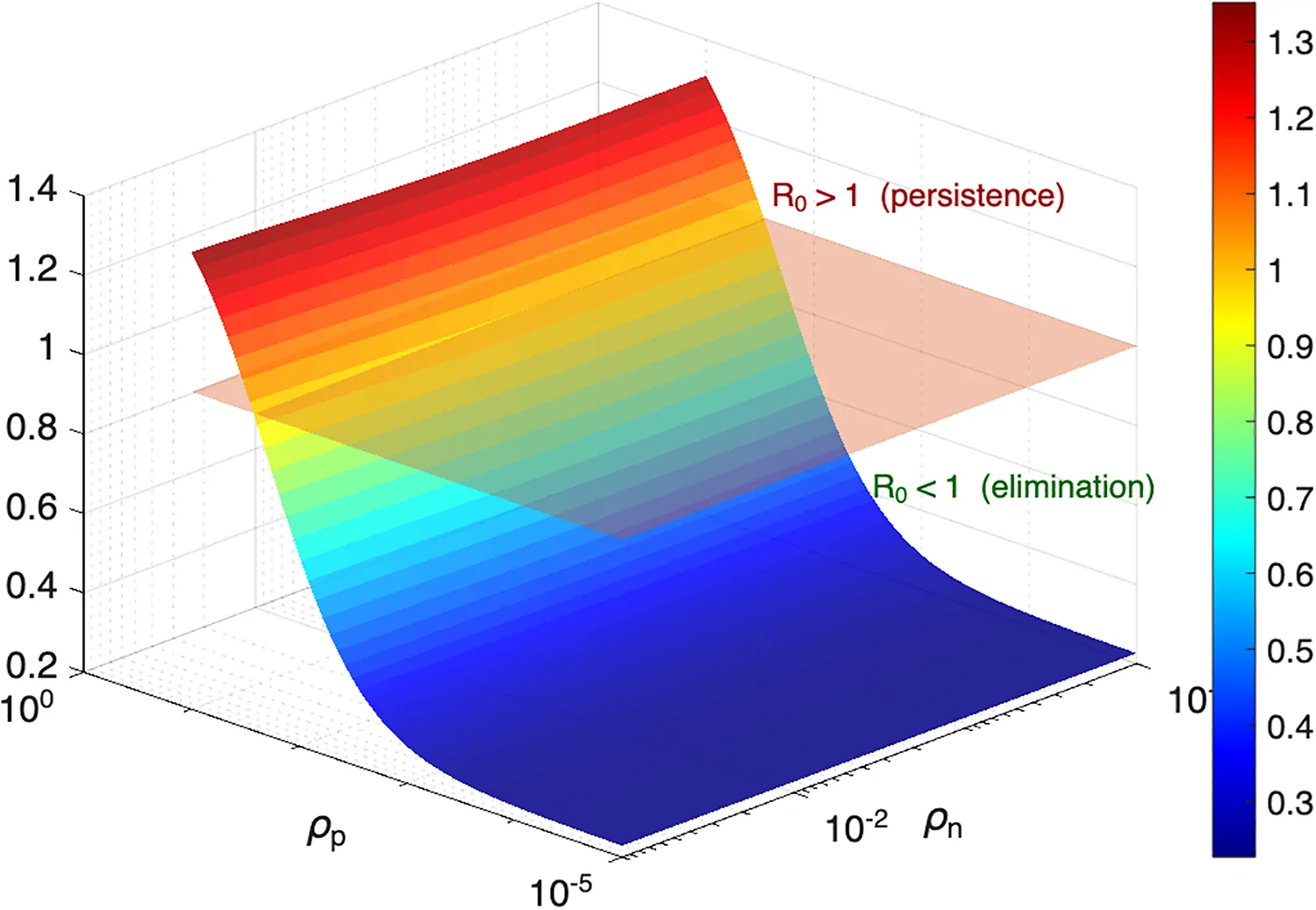
Contour surface of ${ℛ}_{0}$ as a joint function of the custodial petty rehabilitation rate $\rho_{p}$ (left horizontal axis, logarithmic scale) and the custodial serious rehabilitation rate $\rho_{n}$ (right horizontal axis, logarithmic scale). The ${ℛ}_{0}=1$ plane partitions the space into an upper crime-persistence region (${ℛ}_{0}>1$, shaded red) and a lower crime-elimination region (${ℛ}_{0}<1$, shaded blue). ${ℛ}_{0}$ decreases steeply with $\rho_{p}$, confirming that custodial petty rehabilitation is the primary lever for crime elimination, while the surface is comparatively flat with respect to $\rho_{n}$ (serious offenders are a minority, *q* = 0.75).

Fig 13 displays the contour surface of ${ℛ}_{0}$ as a joint function of the two custodial rehabilitation rates, identifying the rehabilitation frontier that separates crime-persistence from crime-elimination regimes.

## Conclusion

This study developed and analysed an eight-compartment ordinary differential equation model to examine the criminogenic effects of incarcerating petty offenders in South Africa. A central structural innovation is the placement of the non-custodial sentencing class $A_{p}$ within a distinct community-supervision stratum that is excluded from the force of crime $\lambda =\beta (C_{p}+\eta C_{n})$, reflecting reduced peer-contagion under supervised diversion. The introduction of the crime generation number ${ℛ}_{0}$, the carceral-induced criminality ratio (CCR, $\Phi_{pn}^{\left(a\right)}$), and the community-supervision escalation ratio (CSER, $\Psi_{pn}$) provides quantitative metrics for evaluating crime persistence and escalation across sentencing regimes. The model was parameterised using South African data from 1995–2024, calibrated to incarceration trends, and validated via global sensitivity analysis.

Results demonstrate that indiscriminate custodial sentencing of petty offenders can amplify criminal activity. The CCR quantifies the probability that custody induces escalation from petty to serious crime through the pathway $J_{p}\to R_{n}\to C_{n}$. The CSER captures the analogous but markedly smaller risk along the diversion route $A_{p}\to R_{p}\to C_{n}$, with the empirical ordering $\Psi_{pn}\ll \Phi_{pn}^{\left(a\right)}$ confirming the substantially lower escalation risk of community supervision. These findings support expanded non-custodial sentencing combined with structured rehabilitation. However, unless the cross-class relapse rate $\omega_{pn}$, the principal driver of the CSER, is reduced, diversion alone may sustain escalation risk, necessitating targeted post-diversion supervision. Short-term interventions increasing κ and $\rho_{a}$ should therefore be complemented by medium-term improvements in sentencing and relapse rates (Table 3).

Two structural simplifications merit attention. First, the omission of pre-trial detention likely leads to underestimation of both ${ℛ}_{0}$ and $\Phi_{pn}^{\left(a\right)}$, as an explicit pre-trial compartment would extend criminal career duration. Second, excluding direct transitions from $J_{p}$ to $C_{n}$ suppresses an additional escalation pathway that would increase $R_{0}^{c_{n}}$ and further amplify the CCR.

The model assumes homogeneous mixing and excludes trial delays and explicit socioeconomic drivers of crime. Given South Africa’s high inequality, unemployment, and concentrated poverty, crime-correlated structural factors could be incorporated through a time-varying recruitment rate, such as $\Lambda (t)=\Lambda_{0}(1+\gamma U(t))$. The homogeneous mixing assumption is most appropriate for dense urban settings like Gauteng, for which the model is explicitly calibrated. Additionally, allocating all released petty offenders to a high-risk class may overstate escalation; allowing a probabilistic return to the petty-risk class would refine CCR estimates. Reliance on literature-based parameter estimates introduces uncertainty, and the framework does not capture intergenerational effects or stigma-related reintegration barriers.

Future work could incorporate stochastic or agent-based formulations to capture individual heterogeneity, as well as socioeconomic covariates to enhance realism. Optimal control methods would enable cost-effectiveness analysis of interventions, while fractional-order extensions could model memory effects in recidivism. Multi-patch structures capturing urban–rural heterogeneity are another important extension. In conclusion, this study advances a rigorous, policy-relevant framework for quantifying the unintended criminogenic consequences of incarcerating petty offenders. The three threshold metrics, ${ℛ}_{0}$, the CCR $\Phi_{pn}^{\left(a\right)}$, and the CSER $\Psi_{pn}$, jointly characterise crime generation and escalation risks across custodial, non-custodial, and community-supervision pathways, enabling pathway-level evaluation previously absent from the literature. Evidence-based policies prioritising non-custodial rehabilitation, relapse prevention, and prison reform are essential for reducing ${ℛ}_{0}$ below unity, curbing recidivism, and achieving durable crime reduction.

## Appendix A: Parameter estimation details

This appendix provides the detailed data sources and statistical justifications for the key parameter choices used in the main text. Table 5 presents the percentage §§of recorded cases for each offence category.

**Table 5:** Extract from SAPS Annual Crime Statistics 2022/2023 [1].

Offence category	Percentage of recorded cases
Theft (general)	32%
Burglary (residential & non-residential)	18%
Shoplifting	8%
Possession of stolen property	5%
Other petty offences	12%
**Total petty offences**	**75%**

The value *q* = 0.75 (proportion of new recruits entering petty crime) is taken as the upper bound of the petty offence share in Gauteng, where property crime prevalence is highest. The calibration of β to achieve $C_{p}^{*}/N^{*}\approx 0.60$ reflects SAPS data indicating that petty offences constitute roughly 60–75% of recorded cases; a conservative midpoint of 0.60 was adopted.

The sentencing rates $\sigma_{p}$ and $\sigma_{n}$ are derived from SALRC (2020) data on average case processing times in Magistrates’ and High Courts (2 years for petty offences, 3 years for serious offences). The non-custodial sentencing proportion κ=0.4 is taken from the JICS Annual Report 2020/2021, which records 35–45% non-custodial disposals for petty offences [30]. The remaining parameters on rehabilitation and relapse, together with their respective justifications, are given in Table 6

**Table 6:** Justification for rehabilitation and relapse parameters.

Parameter	Value	Source & justification
$\rho_{a}$	0.6 yr^-1^	DCS community corrections data [29]; Muntingh [28] reports high success rates for non-custodial diversion.
$\rho_{p}$	0.3 yr^-1^	DCS offender management statistics [29]; Singh [39] estimates ≈30% rehabilitation success for petty offenders in custody.
$\rho_{n}$	0.1 yr^-1^	Singh [39]; lower rehabilitative efficacy for serious offenders.
$\omega_{p}$	0.2 yr^-1^	Samuels et al. [32]: two-year recidivism rate ≈20% for property offenders.
$\omega_{n}$	0.35 yr^-1^	Lötter [33]: two-year recidivism rate ≈35% for violent offenders.

## Appendix B: Equivalence of the geometric loop approach and the next-generation matrix

We establish that the ${ℛ}_{0}$ derived in the main text via the geometric loop approach coincides with the spectral radius of the standard next-generation matrix (NGM) [34–36]. Because $A_{p}$ is excluded from the force of crime $\lambda =\beta (C_{p}+\eta C_{n})$, it does not appear in the new-infection matrix ℱ below. It does appear in the transition matrix 𝒱 (as a draining outflow from $C_{p}$ and a feeding inflow to $R_{p}$). This is structurally consistent with the NGM framework: $A_{p}$ is a *transition* compartment, not an *infection* compartment, and its role is to channel petty offenders through a rehabilitation pathway that carries lower escalation risk than the custodial route.

Following van den Driessche and Watmough [36], we let $𝐱=(C_{p},C_{n},A_{p},J_{p},J_{n},R_{p},R_{n}{)}^{T}$ denote the vector of criminal compartments, ordered so that the two infection (crime generation) classes $C_{p}$ and $C_{n}$ appear first. The linearisation of system (3)–(10) at the CFE ${ℰ}_{0}$ takes the form $\dot{𝐱}=(ℱ-𝒱)𝐱$, where ℱ collects the rates of new crime generation and 𝒱 collects all remaining (transition and death) rates. With $S_{0}=\Lambda /\mu$, these matrices are:


$$\begin{array}{ccc} ℱ & = & (\begin{array}{ccccccc} \beta qS_{0} & \beta q\eta S_{0} & 0 & 0 & 0 & 0 & 0\\ \beta (1-q)S_{0} & \beta (1-q)\eta S_{0} & 0 & 0 & 0 & 0 & 0\\ 0 & 0 & 0 & 0 & 0 & 0 & 0\\ 0 & 0 & 0 & 0 & 0 & 0 & 0\\ 0 & 0 & 0 & 0 & 0 & 0 & 0\\ 0 & 0 & 0 & 0 & 0 & 0 & 0\\ 0 & 0 & 0 & 0 & 0 & 0 & 0 \end{array}), \end{array}$$

and


$$\begin{array}{ccc} 𝒱 & = & (\begin{array}{ccccccc} Q_{1} & 0 & -\kappa \sigma_{p} & 0 & 0 & -\omega_{pp} & -\omega_{np}\\ 0 & Q_{2} & 0 & 0 & -\sigma_{n} & -\omega_{pn} & -\omega_{nn}\\ 0 & 0 & Q_{3} & 0 & 0 & 0 & 0\\ 0 & 0 & 0 & Q_{4} & 0 & 0 & 0\\ 0 & 0 & 0 & 0 & Q_{5} & 0 & 0\\ 0 & 0 & -\rho_{a} & -(1-\pi )\rho_{p} & 0 & Q_{6} & 0\\ 0 & 0 & 0 & -\pi \rho_{p} & -\rho_{n} & 0 & Q_{7} \end{array}). \end{array}$$

The NGM is $K=ℱ{𝒱}^{-1}$. Since ℱ has rank one, *K* has at most one nonzero eigenvalue. The spectral radius is therefore equal to tr(K), which a direct computation yields as


$$\begin{array}{c} \rho (K)=\frac{\beta qS_{0}}{Q_{1}(1-\Phi_{1})}+\frac{\beta \eta S_{0}}{Q_{2}(1-\Phi_{2})}\left[(1-q)+\frac{q\;\Phi_{pn}}{1-\Phi_{1}}\right]={ℛ}_{0}, \end{array}$$

which coincides exactly with expression (35). This confirms that the geometric loop approach and the NGM method are equivalent.
